# Rhodium-catalyzed transformations of diazo compounds *via* a carbene-based strategy: recent advances

**DOI:** 10.1039/d4ra07010k

**Published:** 2024-12-12

**Authors:** Fatemeh Doraghi, Parsa Baghershahi, Mehran Ghasemi, Mohammad Mahdavi, Ahmed Al-Harrasi

**Affiliations:** a Endocrinology and Metabolism Research Center, Endocrinology and Metabolism Clinical Sciences Institute, Tehran University of Medical Sciences Tehran Iran momahdavi@tums.ac.ir; b Pharmaceutical and Heterocyclic Chemistry Research Laboratory, Department of Chemistry, Iran University of Science and Technology Tehran Iran; c Natural and Medical Sciences Research Center (NMSRC), University of Nizwa Nizwa 616 Sulanate of Oman

## Abstract

Diazo compounds are known to be good coupling partners in the synthesis of heterocycles, carbocycles and functionalized molecules *via* a rhodium carbene-based strategy. Many heterocyclic and carbocyclic compounds, including isoquinolones and isocoumarins, quinoxalines, indoles, pyrrones, benzothazines, enaminones, benzenes and seven-membered rings, can be constructed using this rhodium-catalyzed system. The reaction mechanism involves C–H activation, carbene insertion and an annulation/functionalization sequence. This review describes the progress made in the last five years in rhodium-catalyzed transformations of diazo compounds as easily accessible precursors in organic chemistry.

## Abstract 2

As a carbene precursor, diazo compounds incorporate in Rh-catalyzed synthesis of heterocycles, carbocycles and functionalized compounds. The reaction involves C–H activation, carbene insertion and an annulation/functionalization sequence.

## Graphical abstract

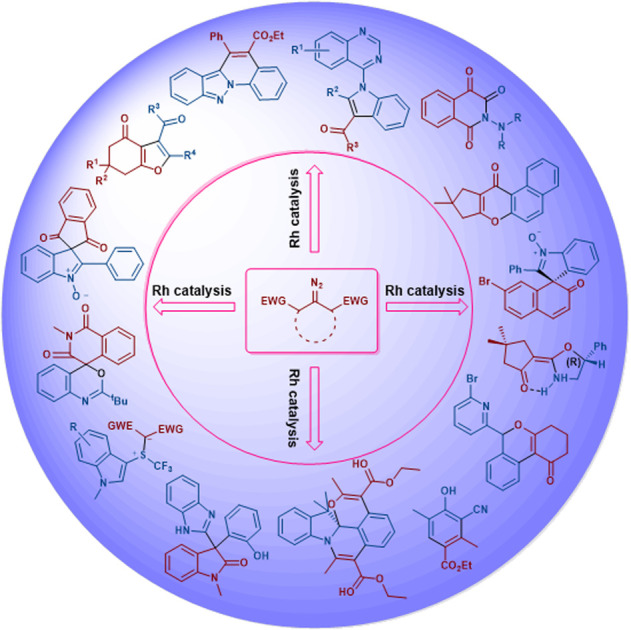

## 1 Introduction

Diazo compounds are an important class of nitrogen-containing compounds that can be used as coupling partners and can be easily accessed from the reaction of cyclic 1,3-diketones and arylsulfonyl azides.^1,2^ Polarized diazo compounds form highly reactive carbene species *via* an interaction with the empty orbital of a transition metal. These carbene species can be involved in a broad range of reactions for the construction of complicated fused and spiro polycyclic frameworks as well as multifunctional organic molecules.^3–9^

Several metal salts, including rhodium,^10–23^ iridium,^24–26^ ruthenium,^27,28^ silver,^29^ indium,^30^ copper^31^ and palladium^32^ can catalyze the transformations of diazo compounds. Of these transformations, the rhodium-catalyzed C–H activation/annulation of diazo compounds has been extensively investigated. The process includes Rh-carbene formation with N_2_ expulsion, the formation of six- and seven-membered rhodacyclic intermediates, and migratory insertion and reductive elimination, which overall can be considered as formal (3 + 2)-, (4 + 1)- or (4 + 2)-cycloadditions. In such reactions, cyclic and acyclic diazo compounds act as a C1, C2, or even C3 synthons. These reactions can be categorized into three groups: (i) Rh-catalyzed C–H insertion/cascade annulation with diazo compounds; (ii) Rh-catalyzed C–C cross-coupling with diazo compounds; and (iii) Rh-catalyzed N–H insertion/annulation.

**Scheme 1:**
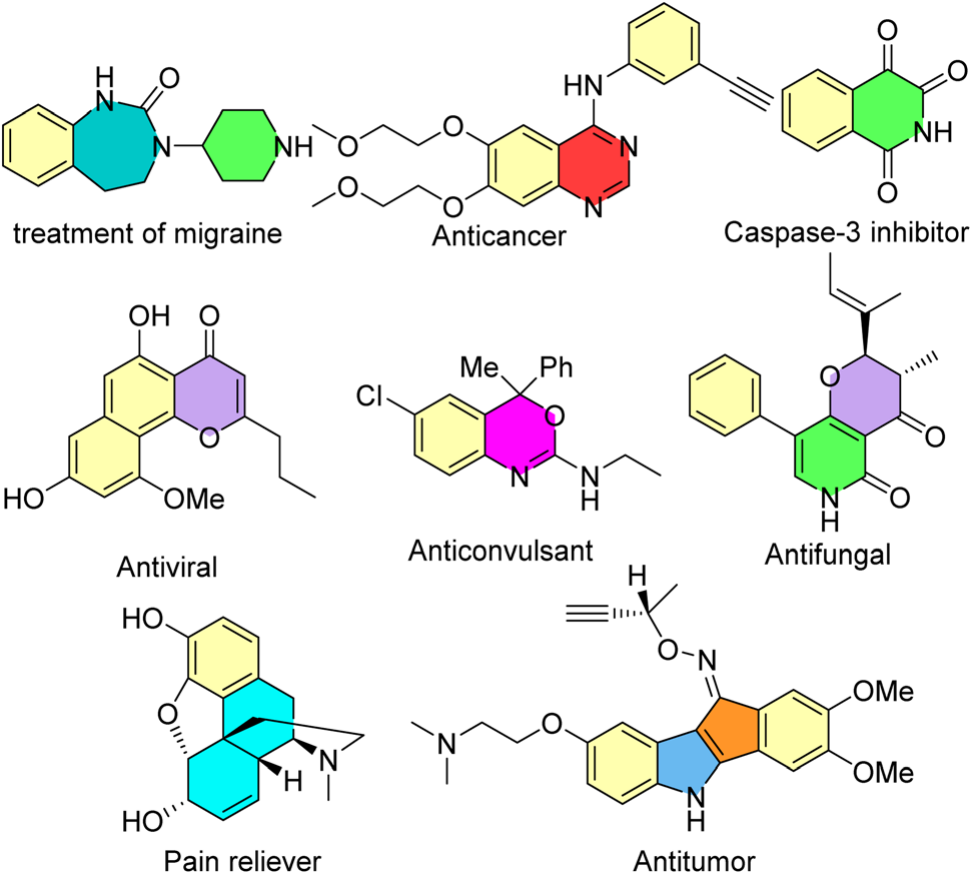
Some biologically active compounds which can be synthesized *via* the rhodium-carbene strategy.

Many heterocyclic and carbocyclic compounds, such as isoquinolones and isocoumarins, quinoxalines, indoles, pyrrones, benzothazines, enaminones, benzenes and seven-membered rings, can be synthesized *via* the rhodium-carbene strategy.^33,34^ Some of these biologically active compounds are shown in Scheme 1.

However, the rhodium-catalyzed selective activation of unactivated C–H bonds generally requires directing groups (DGs) to resolve the regioselectivity. The increased momentum of the carbene insertion makes diazo compounds versatile building blocks,^35,36^ as their uses have expanded to make exciting progress in the field of C–H activation with the help of directing groups (DGs). These directing group strategies fall into two categories: (i) DGs are incorporated into the substrate to aid the site specificity and are then cleaved after activation/functionalization, and (ii) intrinsic DGs in the substrate are used to direct regioselectivity. These DG strategies enhance reaction efficiency and atom economy.

Considering the easy availability and versatility of diazo compounds as carbene sources, and due to the significant role of carbene intermediates in organic chemistry, in this review, we have focused on the progress made in the last five years regarding the transformations of diazo compounds *via* the rhodium-carbene strategy.

## 2 Rhodium-catalyzed transformations of diazo compounds

### 2.1. Synthesis of five-membered rings

**Scheme 2:**
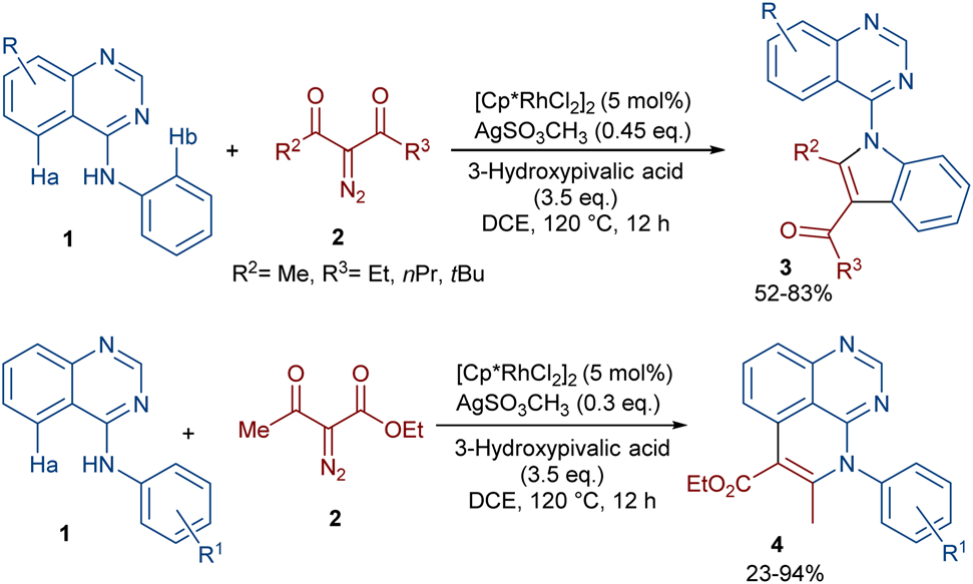
Rh-catalyzed annulation of 4-anilinoquinazolines with diazo compounds.

**Scheme 3:**
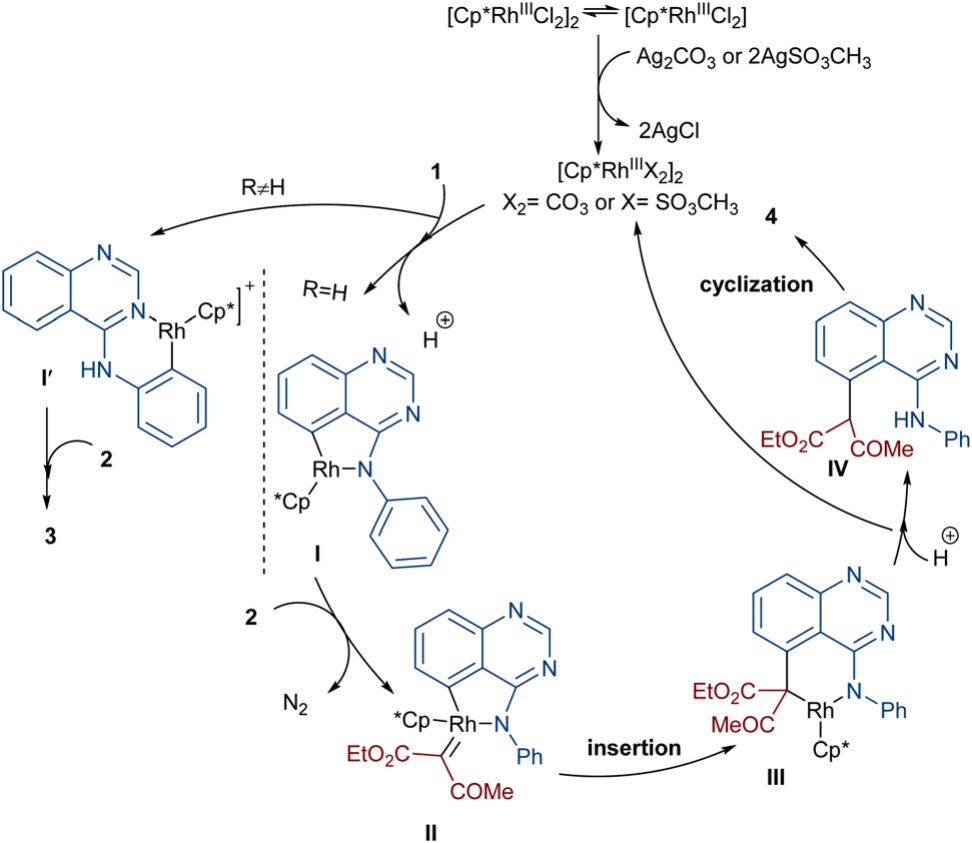
Catalytic cycle for the Rh-catalyzed annulation of 4-anilinoquinazolines with diazo compounds.

In 2020, Dong, Chen and co-workers developed a regioselective strategy for the annulation of 4-anilinoquinazolines with diazo compounds under rhodium catalysis (Scheme 2).^37^ It was proposed that the reaction proceeds through a sequence of C–H activation, carbene insertion and intramolecular cyclization. First, the regioselective C(sp^2^)–H bond activation of substrate 1 gave a Rh-complex I, which in turn coordinated with 2 to generate the Rh-carbene II along with the removal of N_2_. Through migratory insertion and protonolysis, intermediate IV was produced, which underwent intramolecular cyclization to form product 3. When substrate 1 has steric hindrance due to the substituent at the C6 position, the metal center was coordinated with the nitrogen of the pyrimidine, resulting in a six-membered rhodacycle I′ instead of I (Scheme 3). It should be noted that for the formation of compound 4, the optimal oxidant was found to be AgSO_3_CH_3_ instead of Ag_2_CO_3_.

**Scheme 4:**
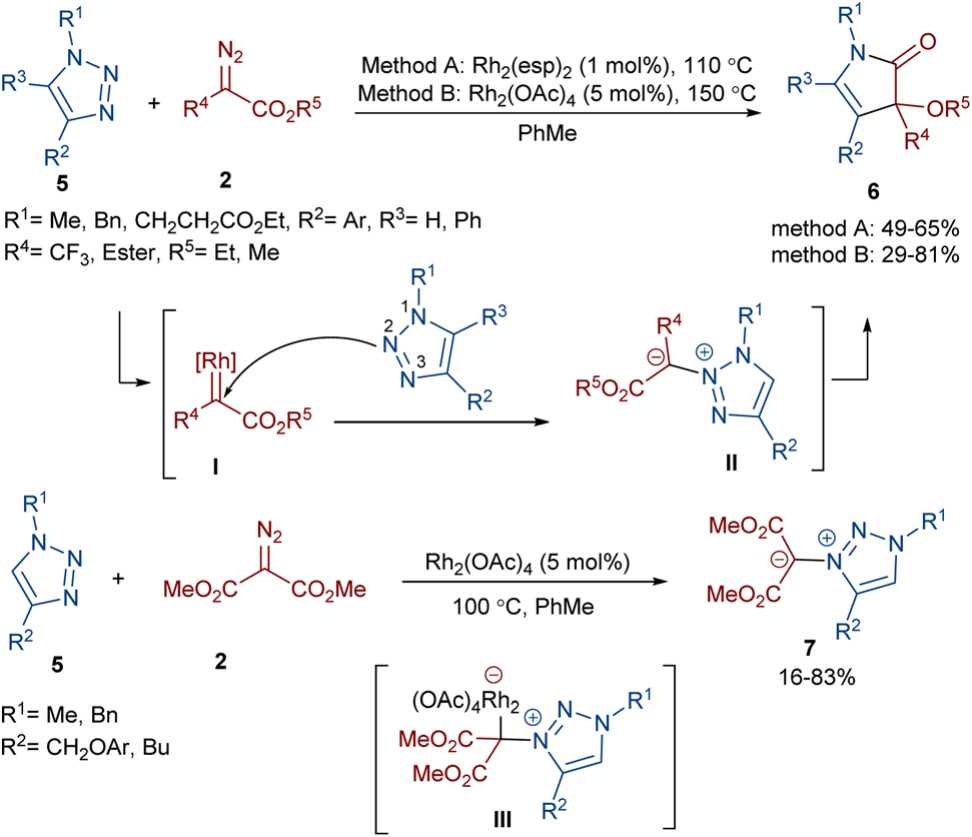
Rh-catalyzed annulation of diazo esters with 1*H*-1,2,3-triazoles.

Two different rhodium(ii) complexes can catalyze the annulation of diazo esters 2 with 1*H*-1,2,3-triazoles 5 (Scheme 4).^38^ In these reactions, various 3-alkoxy-4-pyrrolin-2-one derivatives were constructed in moderate to good yields. First, a Rh-carbenoid intermediate I was formed from the diazo ester and Rh(ii). When R^2^ = aryl, the triazole attacked the electrophilic carbon of I through the N2 atom to give the unstable 3,4-dihydro-1,2,4-triazine II. It seems that the presence of substituents at the C4 position of the triazole shield the N3 center, leading to the regioselective addition of the carbenoid to the N2 center. After that, the pyrrolinone product 6 was obtained *via* the ring contraction of II under the influence of rhodium along with the release of N_2_. However, if R^2^ = alkyl, the attack of the N3 of the triazole on the carbenoid proceeded *via* rhodium intermediate III affording 1,2,3-triazol-3-ium ylides 7. DFT calculations confirmed these results by showing a 3.2 kcal mol^−1^ decrease in the free energy of complex III compared to II when R^2^ was alkyl not aryl, which is because of the absence of conjugation between the aryl and triazole rings.

**Scheme 5:**
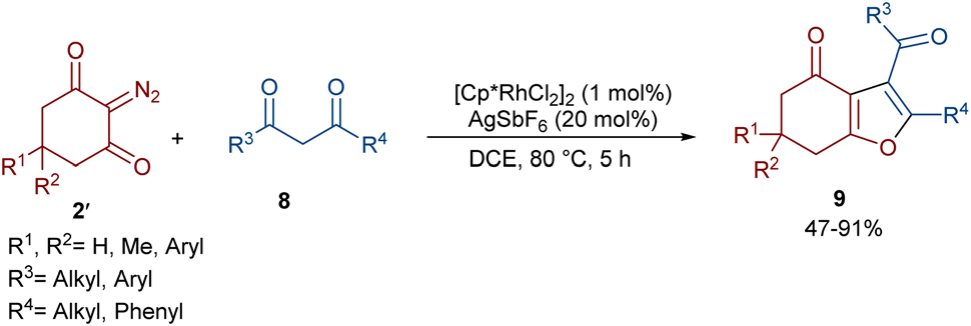
Rh-catalyzed annulation of cyclic 2-diazo-1,3-diketones with β-keto esters.

**Scheme 6:**
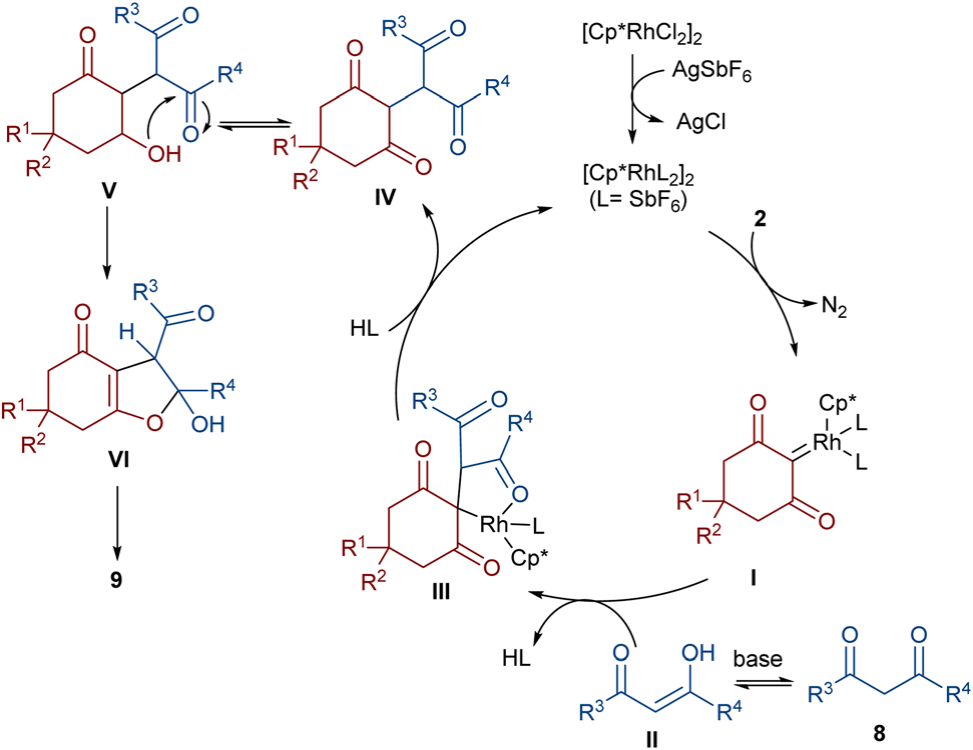
Possible catalytic cycle for the Rh-catalyzed annulation of cyclic 2-diazo-1,3-diketones with β-keto esters.

**Scheme 7:**
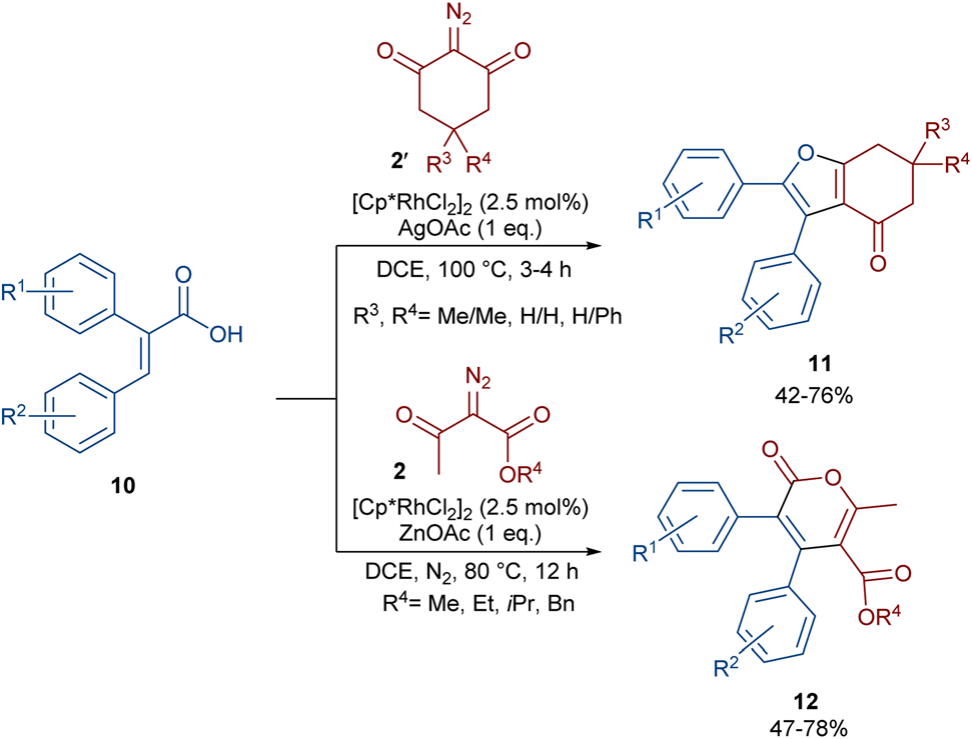
Rh-catalyzed annulation of *cis*-stilbene acids with diazo compounds.

**Scheme 8:**
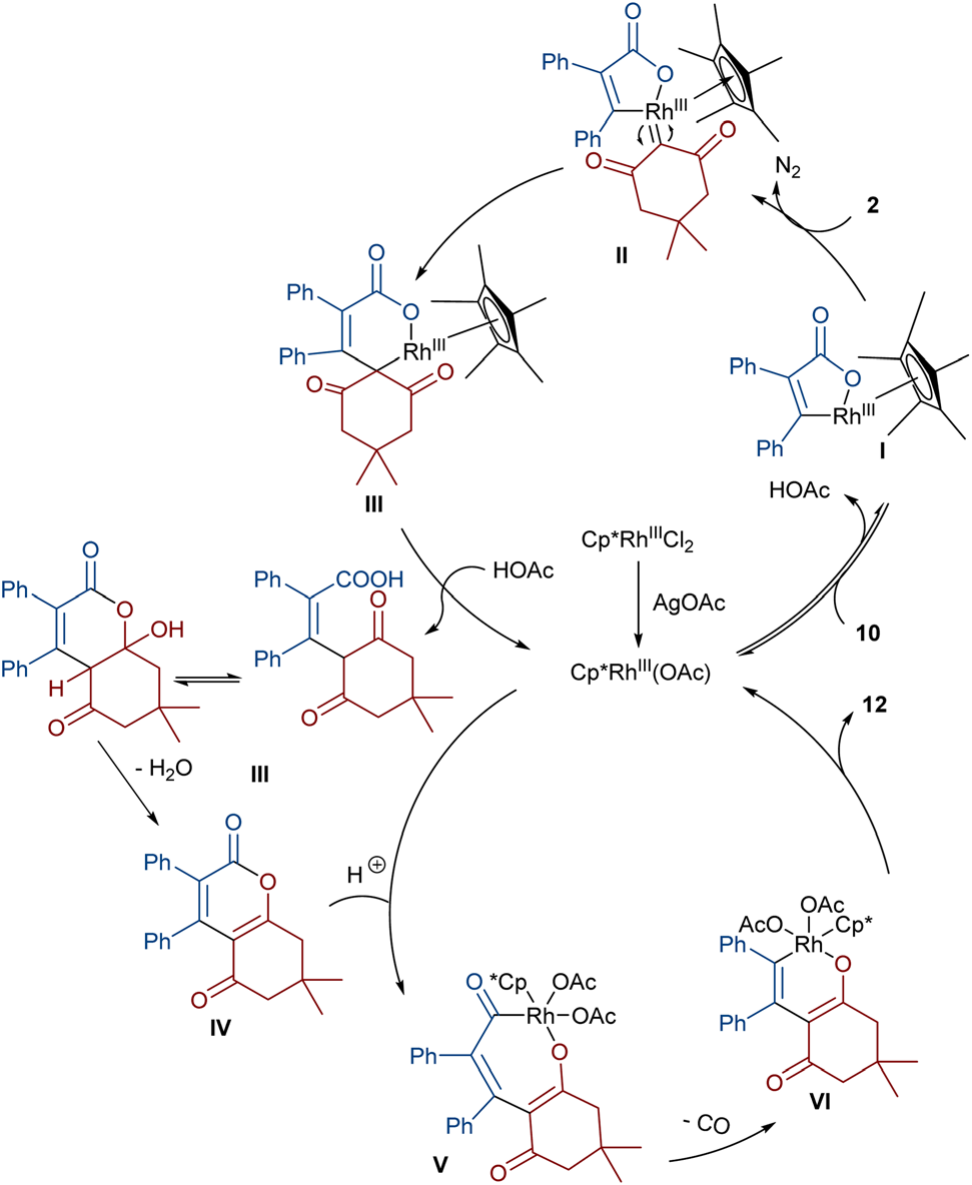
Possible mechanism for the Rh-catalyzed annulation of *cis*-stilbene acids with diazo compounds.

In 2021, Shang and co-workers used a rhodium catalyst to make a furan ring *via* the annulation of 2-diazo-1,3-diketones 2 with 1,3-dicarbonyl compounds 8 (Scheme 5).^39^ The reaction mechanism was rationalized with the help of H/D exchange and kinetic isotope effect (KIE) experiments. The rate-determining step was predicted to be the C–H activation step. A ligand exchange between the pre-catalyst [Cp*RhCl_2_]_2_ and AgSbF_6_ occurred to obtain an active catalyst [Cp*Rh(SbF_6_)_2_]_2_, which in turn reacted with 2 to form Rh-carbene I with the release of N_2_. Meanwhile, the enolization of 1,3-diketones 8 resulted in intermediate II, which attacked I to form intermediate III. Finally, product 9 was achieved through a sequence of protonolysis, enol-ketone tautomerization, intramolecular cyclization and dehydration (Scheme 6). A gram-scale synthesis (0.89 g, 86%) and the post-functionalization of the obtained products in the presence of N_2_H_4_·H_2_O, NH_2_OH·HCl, or NaBH_4_ were also performed. Another work on the synthesis of furan structures starting from diazo compounds was reported in 2024 (Scheme 7).^40^ By changing some parameters, like additive and temperature in the reactions of *cis*-stilbene acids 10 and 2-diazo-1,3-diketones 2, Shankaraiah and co-workers could isolate two different annulated products, 6,7-dihydrobenzofuran-4(5*H*)-ones 11 and α-pyrones 12. Both products were produced *via* a metal carbene strategy, in which two migratory insertions led to dihydrofuran formation. The mechanism involved the formation of the active catalyst from a rhodium pre-catalyst and Lewis acid, followed by the reaction with *cis*-stilbene acid to form rhodacycle I. After the formation of carbene II from the diazo compounds, and sequential migratory insertion and protonation, intermediate IV was furnished. The second migratory insertion of Rh(iii) provided intermediate V with the removal of CO. Finally, product 12 was furnished *via* reductive elimination (Scheme 8).

**Scheme 9:**
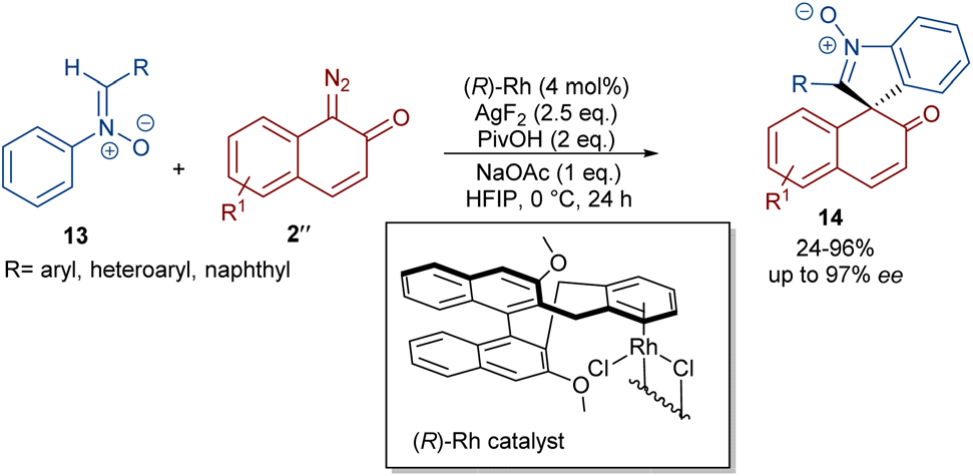
Rh-catalyzed reaction of diazo compounds and nitrones.

**Scheme 10:**
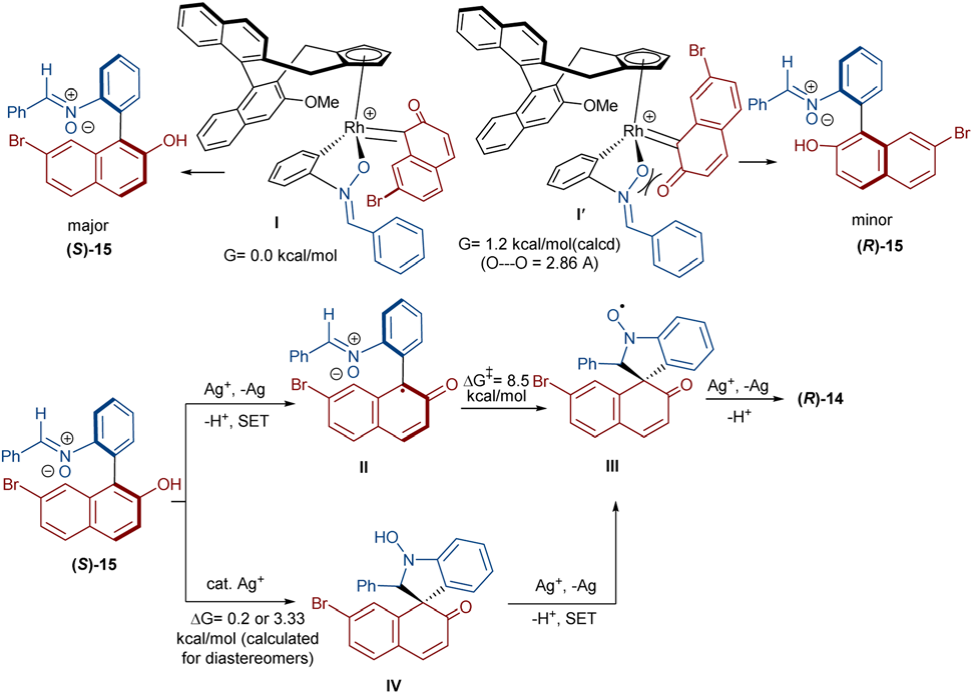
Rational mechanism for the Rh-catalyzed reaction of diazo compounds and nitrones.

**Scheme 11:**
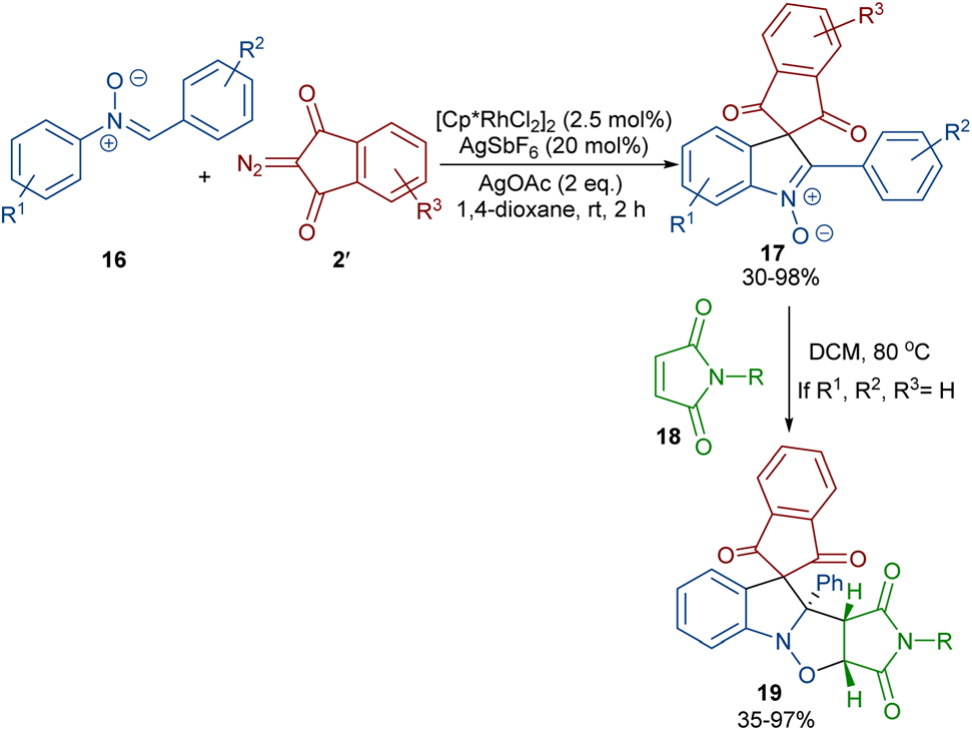
Rh-catalyzed reaction of *N*-aryl nitrones and 2-diazo-1,3-indandiones.

**Scheme 12:**
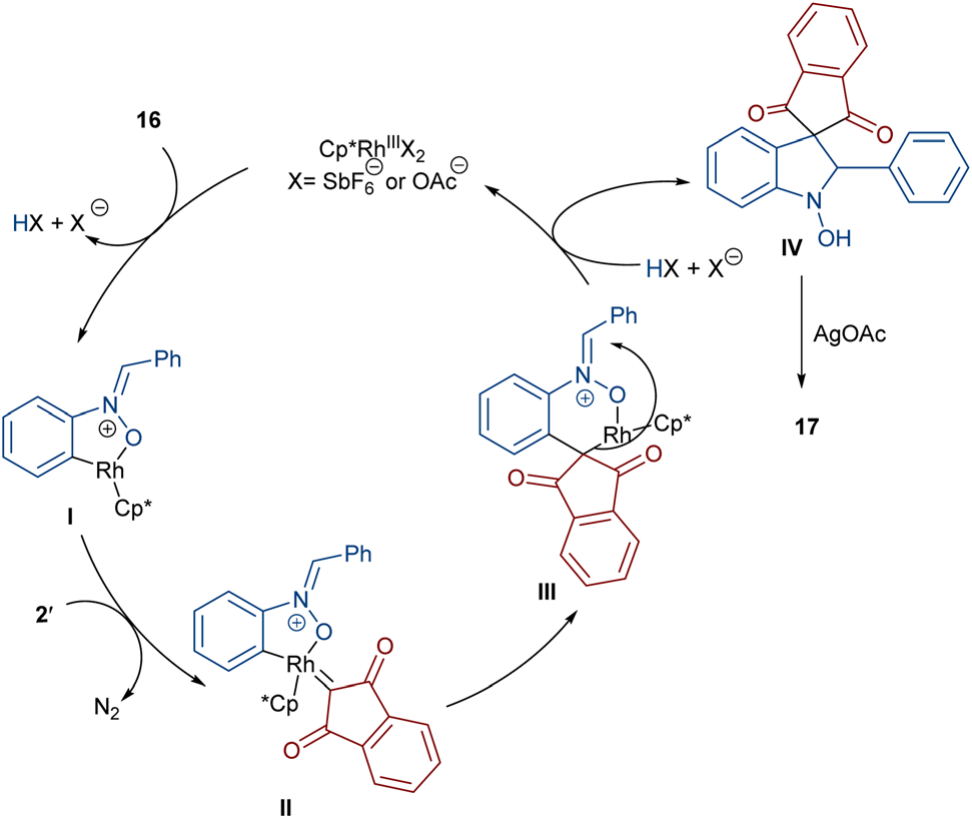
Proposed mechanism for the Rh-catalyzed reaction of *N*-aryl nitrones and 2-diazo-1,3-indandiones.

In 2020, Li and his team developed an enantioselective synthesis of spirocyclic compounds 14 from quinone diazides 2′′ and nitrones 13 (Scheme 9).^41^ The reaction proceeded through the formation of a rhodacyclic intermediate, which underwent addition of a diazo compound to form a stable biaryl intermediate (*S*)-15*via* transition state I. With the assistance of Ag(i), single electron transfer (SET) oxidation occurred in 15 to give radical II, which rapidly cyclized to generate the more stable nitroxide radical III. A second SET process in the presence of Ag(i) led to spiro-cyclic product 14 (Scheme 10). KIE values revealed that the C–H cleavage was not the rate-determining step. In 2023, Guo *et al.* introduced a new strategy for the synthesis of spiro-cyclic indole-*N*-oxides 17 from *N*-aryl nitrones 16 and 2-diazo-1,3-indandiones 2′ (Scheme 11).^42^ It was found that the presence of [Cp*RhCl_2_]_2_, AgSbF_6_ and AgOAc were all necessary to catalyze this reaction under mild conditions. The reaction proceeded through (4 + 1)-cycloaddition, where the diazo compound acted as a C1 synthon. According to H/D and KIE studies, a possible mechanism was proposed, in which nitrone 16 and Rh produced the five-membered rhodacycle I, which then underwent carbene insertion to give the Rh-carbene II. Next, through a migratory insertion, II was converted to the six-membered rhodacycle III, followed by intramolecular nucleophilic addition and protonation to render spiro-cyclic intermediate IV along with the regeneration of the Rh(iii) species. Finally, IV was oxidized by AgOAc to provide product 17 (Scheme 12). Additionally, (3 + 2)-cycloaddition of the obtained product could be carried out with maleimides 18 to access biologically active maleimide-fused polycyclic compounds 19 in a diastereoselective manner.

**Scheme 13:**
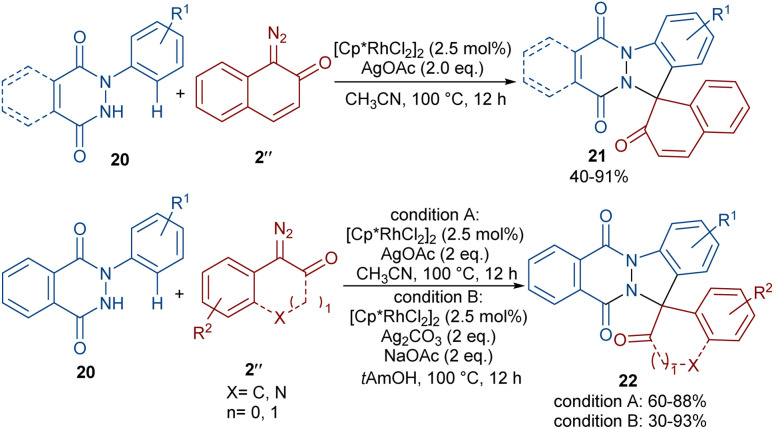
Rh-catalyzed reaction of *N*-aryl phthalazine-diones and diazo compounds.

**Scheme 14:**
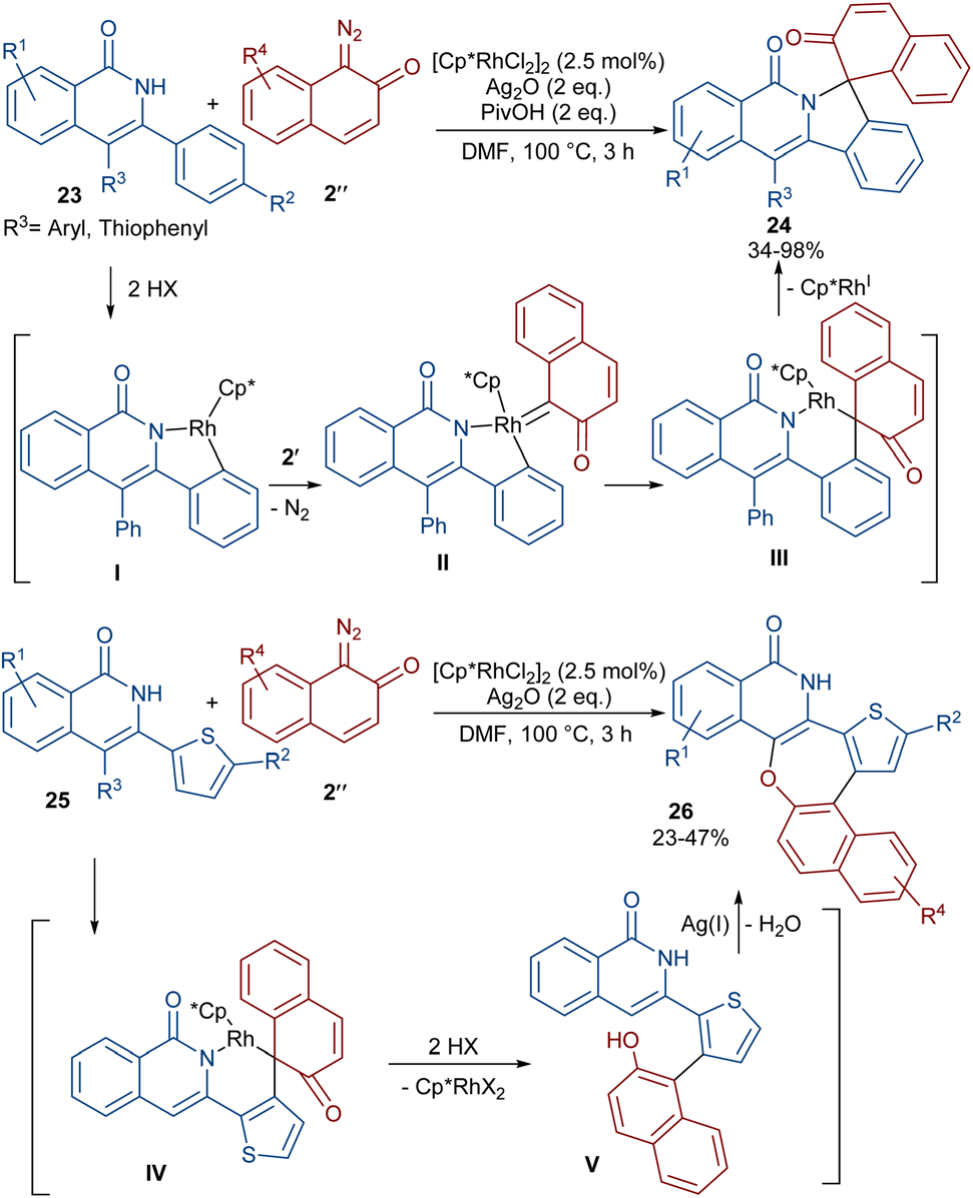
Rh-catalyzed reaction of isoquinolones and diazo compounds.

A (4 + 1)-annulation strategy was reported for the construction of spiro-cyclic indazoles 21 (Scheme 13).^43^ In this regard, *N*-aryl phthalazine-diones 20 were used as a reagent to react with 1-diazonaphthalen-2(1*H*)-one 2′′. This spiro-annulation involved Rh-catalyzed C–H bond activation, carbene insertion and nucleophilic addition. It is noteworthy that other metal catalysts, such as [Cp*IrCl_2_]_2_ and [RuCl_2_(*p*-cymene)]_2_, also gave the desired products in 73% and 25% of yields, respectively. Conversely, [Cp*Co(CO)I_2_]_2_ was not found to be a workable catalyst. A similar (4 + 1)-annulation for the construction of spiro-cyclic compounds under rhodium catalysis was reported by another research team (Scheme 14).^44^ In their work, the reaction was carried out using isoquinolones 23 and 1-diazonaphthalen-2(1*H*)-ones 2′′ as substrates. The cyclization proceeded through intermediate I, where diazo compound 2′′ acted as a C1 synthon. Moreover, when 3-(thiophen-2-yl)isoquinolin-1(2*H*)-one 25 was used as a reactant in the reaction with diazonaphthalen-2(1*H*)-ones 2′′, a series of oxepine-fused polycyclic compounds 26 were synthesized *via* (4 + 3)-annulation, in which the diazo compound acted as a C3 synthon and was incorporated in the formation of the six-membered rhodacycle IV.

**Scheme 15:**
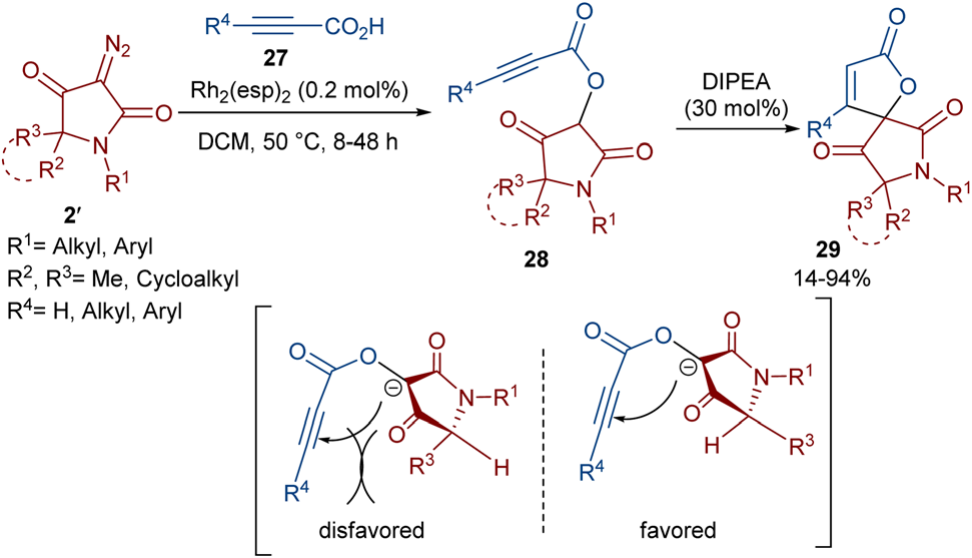
Rh-catalyzed reaction of propiolic acids and diazo compounds.

**Scheme 16:**
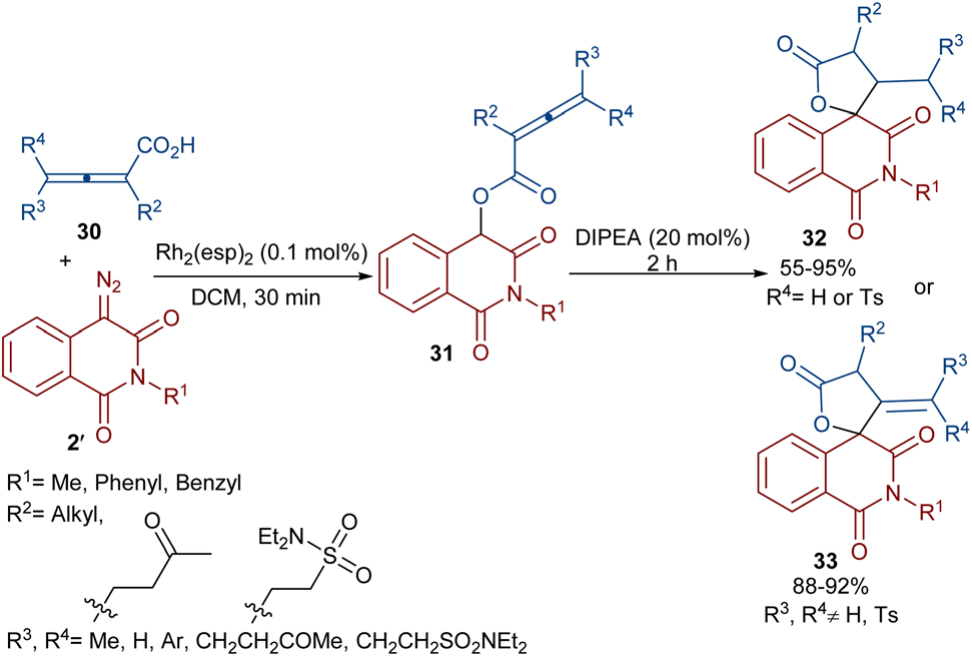
Rh-catalyzed reaction of allenic acids and diazo compounds.

Dar'in *et al.* developed a strategy for the synthesis of spiro-cyclic products 28 from propiolic acids 27 and a new class of diazo compounds 2′ (Scheme 15).^45^ For this purpose, they synthesized the new diazo compounds by the treatment of 3-methoxy-3-oxopropanoic acid and α-amino acid methyl esters under basic conditions. Through a multi-steps process, they achieved 3-diazotetramic acid substrates 28. The reaction of the obtained diazo compounds with propiolic acids proceeded through a Rh-catalyzed carbene formation, followed by O–H insertion into the propiolic acids to yield compound 28. The subsequent base-promoted intramolecular Michael addition from the less hindered side of the molecule furnished the bioactive spirocyclic butenolide products 29 in up to 94% yield. Notably, the position of the hydrogen atom as the R^2^ substituent was important in the diastereoselectivity and the formation of the spirocycle. Again, this group could synthesize spiro-cyclic butenolides using heterocyclic diazo compounds 2′ and allenic acids 30 (Scheme 16).^46^ Two products 32 and 33 can be produced from adduct 31, which in turn was generated from the reaction of allenic acid and a diazo compound under rhodium(ii) catalysis. It was found that the position of the double bond in β-methylidene furanone 33 is due to a tautomerization process. DFT calculations revealed that 33 with an exocyclic double bond is less thermodynamically stable relative to 32. Therefore, 33 can be converted into 32 in the presence of a base.

**Scheme 17:**
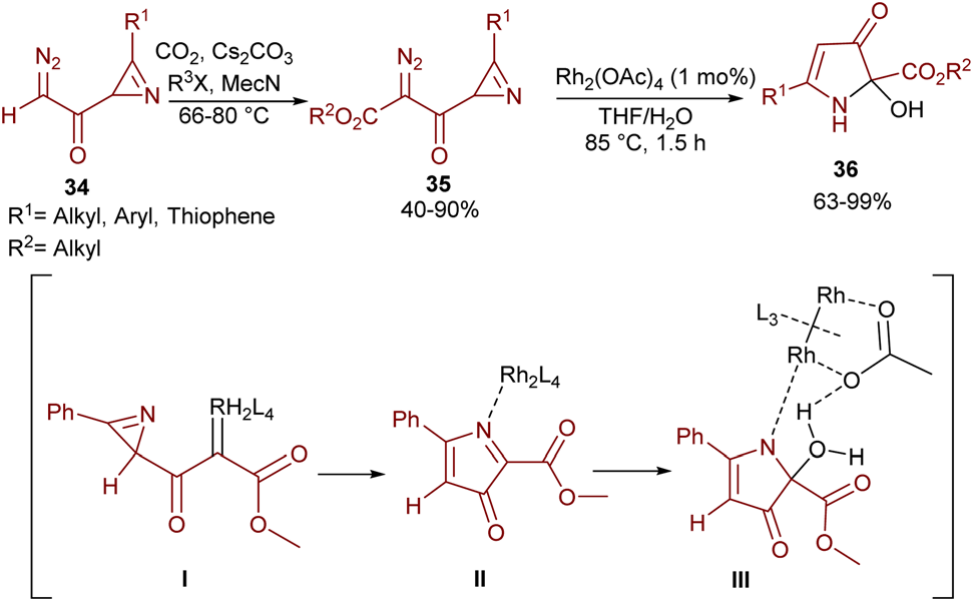
Rh-catalyzed intramolecular azirine ring expansion.

An intramolecular aziridine ring-expansion carbene insertion strategy was reported by Khlebnikov and his team (Scheme 17).^47^ In this regard, azirinyl-substituted diazodicarbonyl compounds 35 were initially prepared from diazoacetylazirines 34 and then successfully used as substrates in the presence of a rhodium(ii) complex to provide 2-hydroxy-3-*oxo*-2,3-dihydro-1*H*-pyrrole-2-carboxylates 36. The source of the hydroxyl group was found to be water. With the help of DFT calculations, the reaction was suggested to proceed through a Rh-carbenoid intermediate I, which under ring-expansion gave pyrrolone complex II. Then, the insertion of H_2_O into the C–N bond and the liberation of Rh afforded the final product 36.

**Scheme 18:**
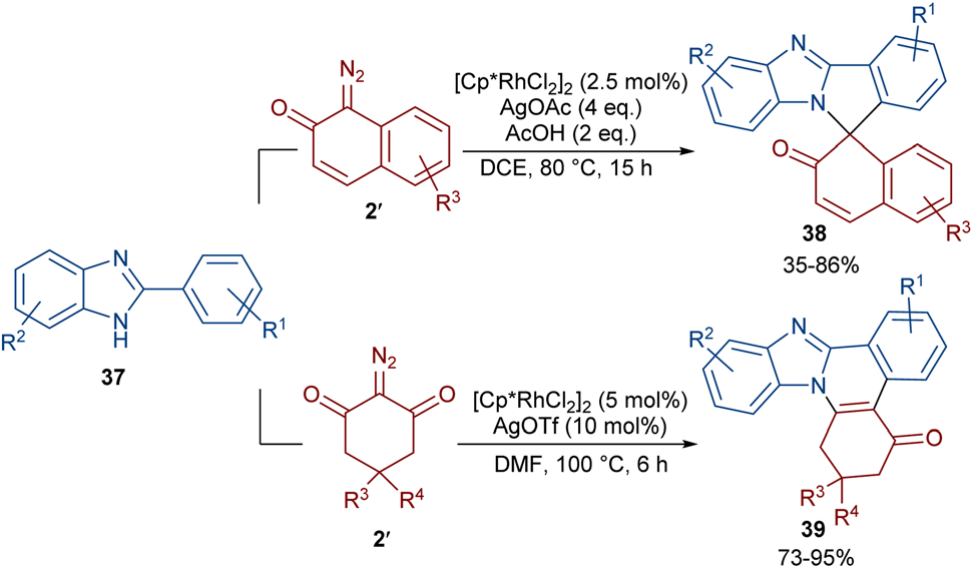
Rh-catalyzed reaction of 2-arylbenzimidazoles and diazo compounds.

Two (4 + 1) and (4 + 2) annulation methodologies were suggested for the reaction of 2-arylbenzimidazoles 37 with diazo compounds 2 (Scheme 18).^48^ Depending on their structure, diazo compounds can act as a C1 and C2 synthon in the cycloaddition with 2-arylbenzimidazoles. The reaction of 2-arylbenzimidazoles with 1-diazonaphthalen-2(1*H*)-one 2′′ proceeded through (4 + 1)-cycloaddition to produce spirocyclic benzimidazole-fused isoindole naphthalen-2-ones 38. This reaction involved C–H activation, Rh-carbene formation, migratory insertion, proto-demetallation and an intramolecular nucleophilic addition of the NH group of the benzimidazole to the carbonyl group, followed by dehydration. Conversely, a (4 + 2)-annulation was involved in the reaction of 2-arylbenzimidazoles and 2-diazocyclohexane-1,3-diones 2′. In this case, benzimidazole-fused quinolines 39 were obtained *via* Rh-carbene formation, migratory insertion and reductive elimination.

### 2.2. Synthesis of six-membered rings

**Scheme 19:**
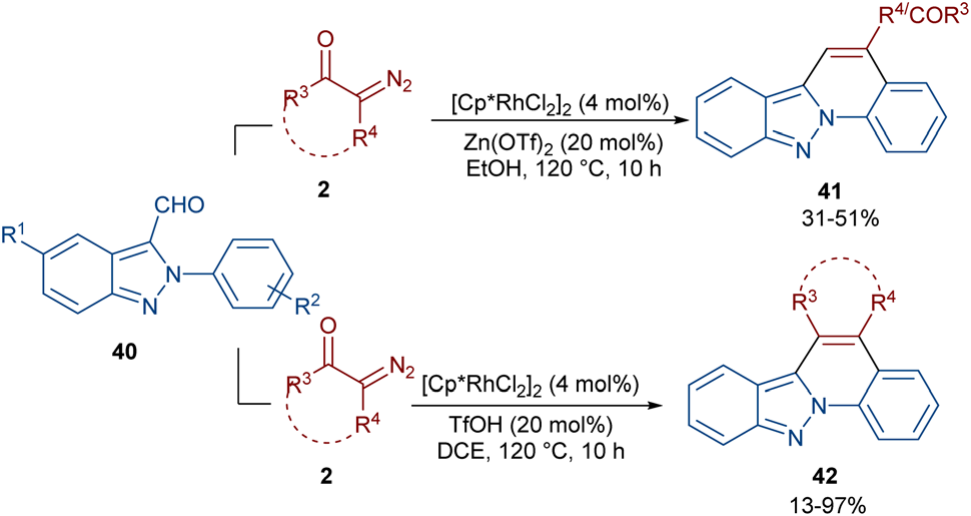
Rh-catalyzed annulation of 2-aryl-2*H*-indazoles with diazo compounds.

**Scheme 20:**
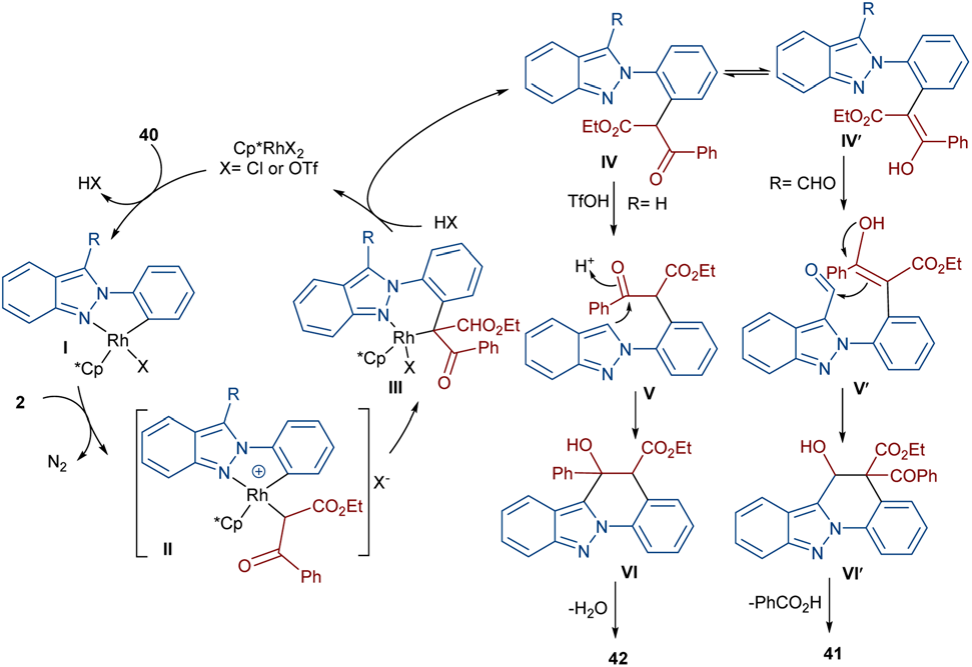
Possible mechanism for the Rh-catalyzed annulation of 2-aryl-2*H*-indazoles with diazo compounds.

In 2020, Guo *et al.* reported the annulation of 2-aryl-2*H*-indazoles 40 with α-diazo compounds 2 to give a quinolone ring in the presence of the same Rh catalyst (Scheme 19).^49^ In this synthetic method, the diazo compound acted as either a C1 or C2 synthon. The use of Zn(OTf)_2_ as a Lewis acid or TfOH as a Brønsted acid together with [Cp*RhCl_2_]_2_ as a catalyst can lead to two different indazole derivatives. The reaction started with a Rh(iii)-catalyzed indazole-assisted C–H bond activation followed by the coordination with diazo compound 2 to obtain the carbene intermediate II. The migratory insertion of II yielded a six-membered rhodacycle III, which under proto-demetallation gave intermediates IV or IV′. In this step, when R = H, IV moved through an intramolecular C3-nucleophilic addition to deliver a six-membered intermediate IV, followed by the H_2_O removal to afford product 40. While, if R = CHO, product 41 was furnished *via* intermediate IV′ through a regioselective nucleophilic attack of the α-carbon on the carbonyl group to give a six-membered intermediate VI′, followed by acid elimination (Scheme 20).

**Scheme 21:**
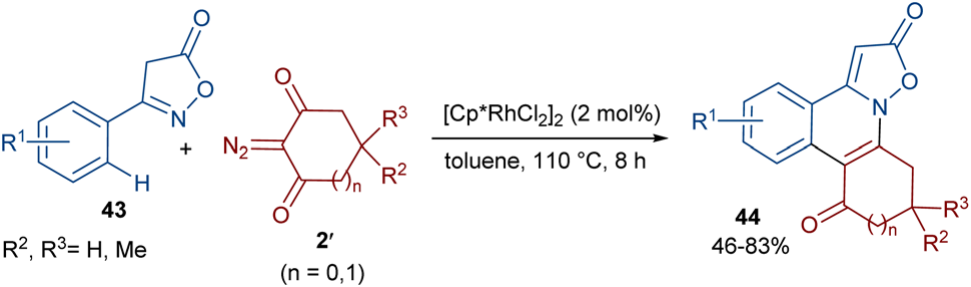
Rh-catalyzed annulation of 3-arylisoxazolones and cyclic 2-diazo-1,3-diketones.

The Shang research team reported the Rh-catalyzed annulation of 3-arylisoxazolones 43 and cyclic 2-diazo-1,3-diketones 2′ (Scheme 21).^50^ A wide range of isoxazolo[2,3-*f*]phenanthridines 44 were prepared in the presence of a low catalytic amount of a Rh(iii) complex (2 mol%) without any additives. To gain a better understanding of the reaction mechanism, the authors designed H/D exchange and KIE experiments. The results of the H/D exchange showed that the C(sp^2^)–H bond activation is fast and no alkenylation occurred on the isoxazol-5(4*H*)-one ring. The following KIE experiments suggested that the C–H activation is the rate-determining step. Thus, the reaction was believed to proceed through sequential Rh-catalyzed C–H activation, Rh-carbene formation, migratory insertion, protonolysis and hydrogen transfer.

**Scheme 22:**
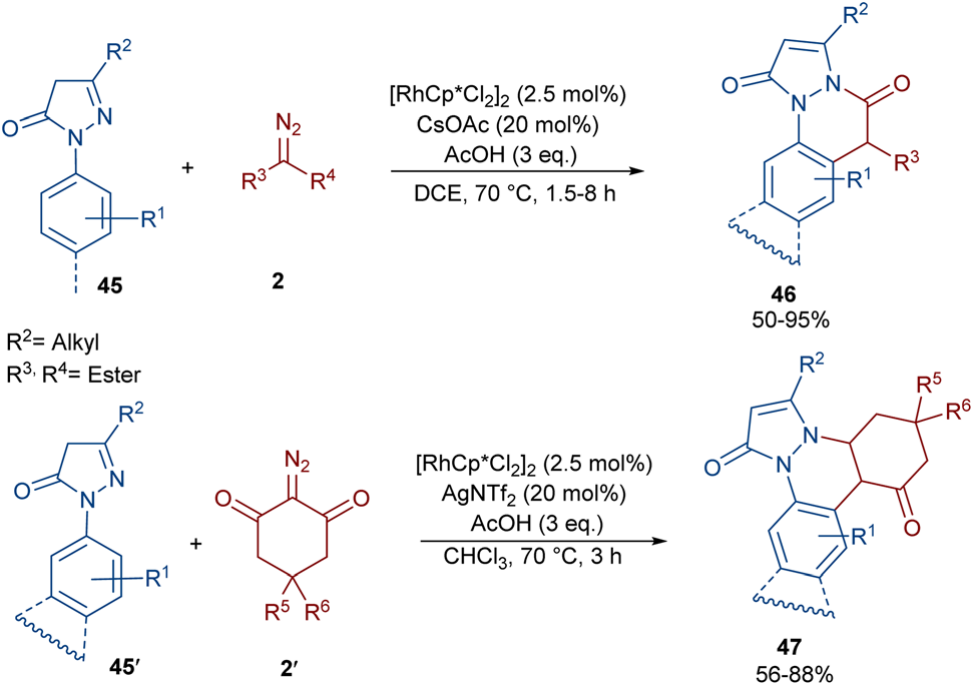
Rh-catalyzed annulation of *N*-aryl pyrazolones with diazo compounds.

Sun and co-workers were able to synthesize a new class of pyrazolone-fused cinnolines 46, 47 through the annulation of *N*-aryl pyrazolones 45, 45′ with diazo compounds 2 (Scheme 22).^51^ The reaction was reported to involve rhodium-carbene formation, migratory insertion and proto-demetallation. A diverse range of α-diazo esters, α-diazo ketones, phosphate diazo compounds, and cyclic diazo compounds were well tolerated as the carbene precursors, leading to the synthesis of dihydropyrazolo[1,2-*a*]cinnolines 46 and dihydrobenzo[*c*]pyrazolo[1,2-*a*]cinnoline-1,8-diones 47. Besides *N*-aryl pyrazolones, an *N*-naphthyl pyrazolone also gave the corresponding product in 80% yield. To reveal the synthetic utility of the method, a gram-scale synthesis (1.46, 94%) and further transformation of the products with the Lawesson reagent as well as a hydrolysis reaction were also performed, resulting in 53% and 70% yields, respectively.

**Scheme 23:**
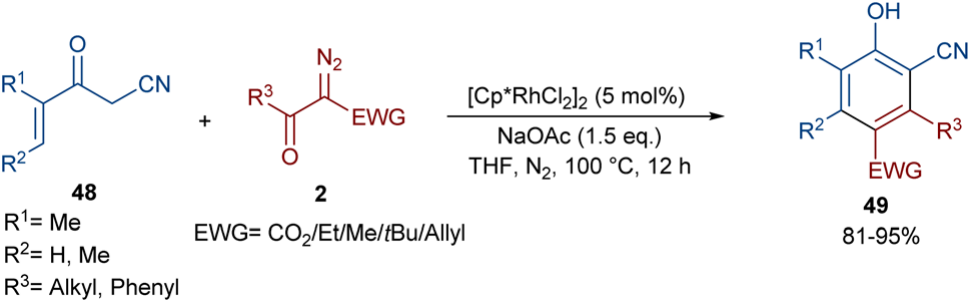
Rh-catalyzed annulation of diazo compounds with 3-oxopent-4-enenitriles.

**Scheme 24:**
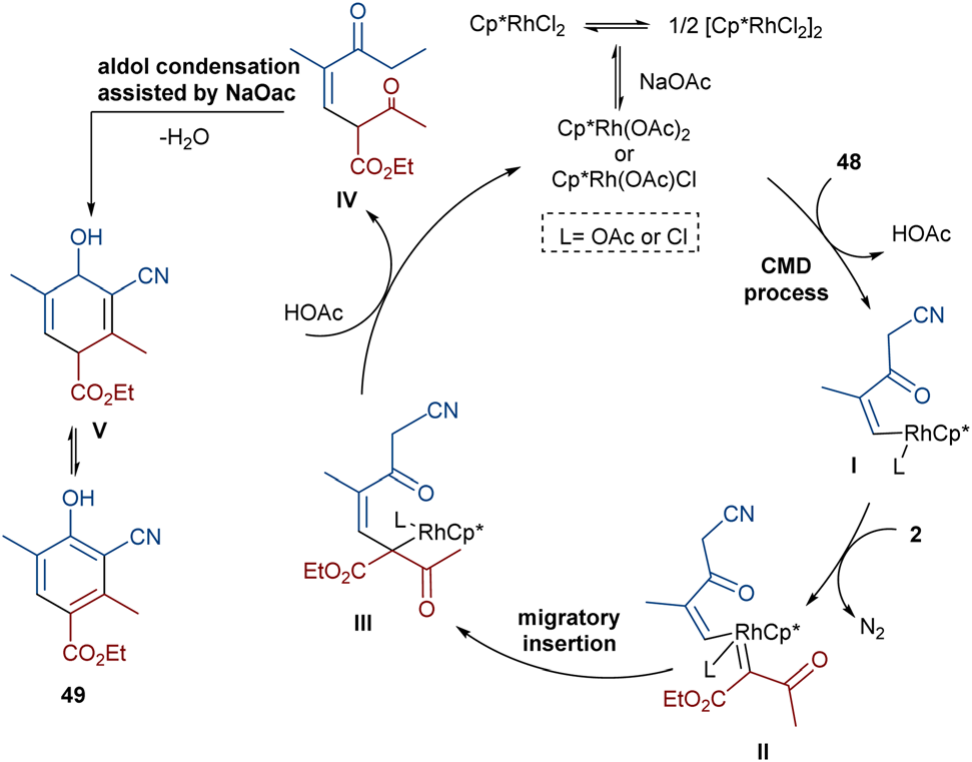
Possible mechanism for the Rh-catalyzed annulation of diazo compounds with 3-oxopent-4-enenitriles.

**Scheme 25:**
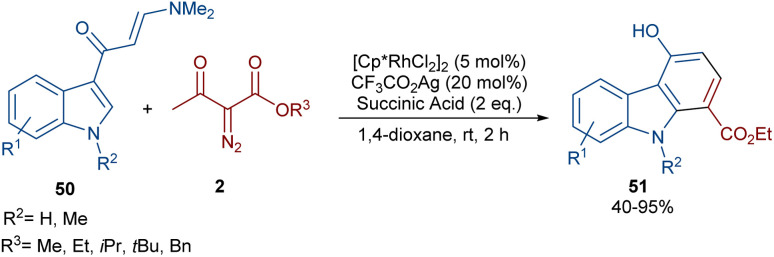
Rh-catalyzed annulation of indole-enaminones with diazo compounds.

**Scheme 26:**
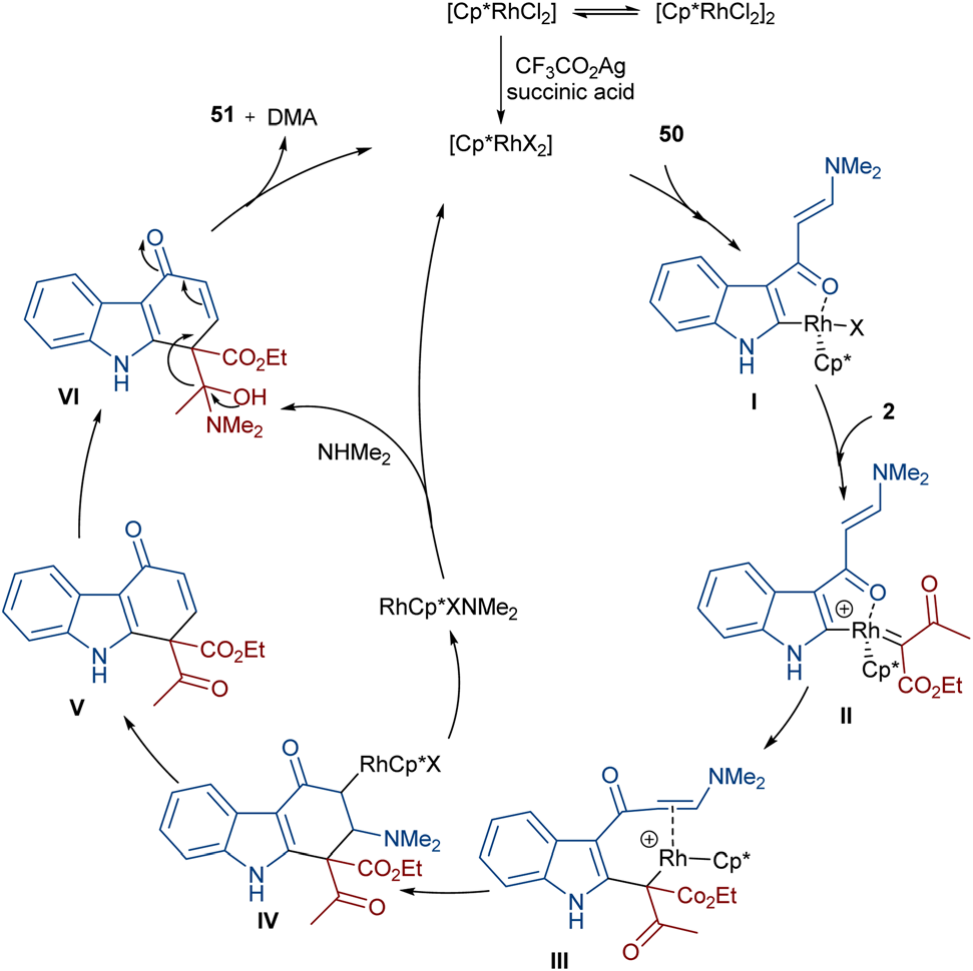
Catalytic cycle for the Rh-catalyzed annulation of indole-enaminones with diazo compounds.

In 2021, Wang and co-workers described a methodology for the (2 + 4)-annulation of diazo compounds with 3-oxopent-4-enenitriles (Scheme 23).^52^ A series of multi-functionalized phenols were well synthesized in high to excellent yields. The reaction commenced with the initial formation of the active catalysts, Cp*Rh(OAc)_2_ or Cp*Rh(OAc)Cl, which participate in the carbonyl-assisted C–H bond cleavage of 48. The obtained intermediate I coordinated with diazo compound 2 to form the Rh-carbene intermediate II. Then, migratory insertion of the carbene into the C–Rh bond gave intermediate III, which converted to intermediate IV through the protonolysis and regeneration of the active Rh catalyst. Subsequently, intramolecular aldol condensation, a double bond shift and aromatization led to product 49 (Scheme 24). It is noteworthy that the base is involved in both the C–H bond activation and the aldol condensation reaction, which justified using a stoichiometric amount of NaOAc. Another synthetic method for the preparation of phenol structures was reported by Liu, Zhou and co-workers (Scheme 25).^53^ For this purpose, they used indole-enaminones 50 and diazo compounds 2 in the presence of a rhodium catalyst. This reaction was carried out *via* an unexpected (5 + 1)-cyclization process instead of the more common (4 + 2)-cyclization. First, through a ligand exchange, the active catalyst [Cp*RhX_2_] was formed, which rapidly reacted with indole-enaminone 50 to form complex I. The coordination of α-diazo-β-ketoester 2 to I resulted in the formation of rhodium carbene II. Sequential intramolecular migratory insertion, and coordination with the alkene bond gave complex III. Then, III converted to the six-membered cyclic intermediate IV*via* migratory insertion and protonolysis, followed by the elimination of the rhodium species to render intermediate V. The further elimination of *N*,*N*-dimethylamine led to VI. After that, *N*,*N*-dimethylacetamide was removed from IV to be aromatized into the desired product 51 (Scheme 26).

**Scheme 27:**
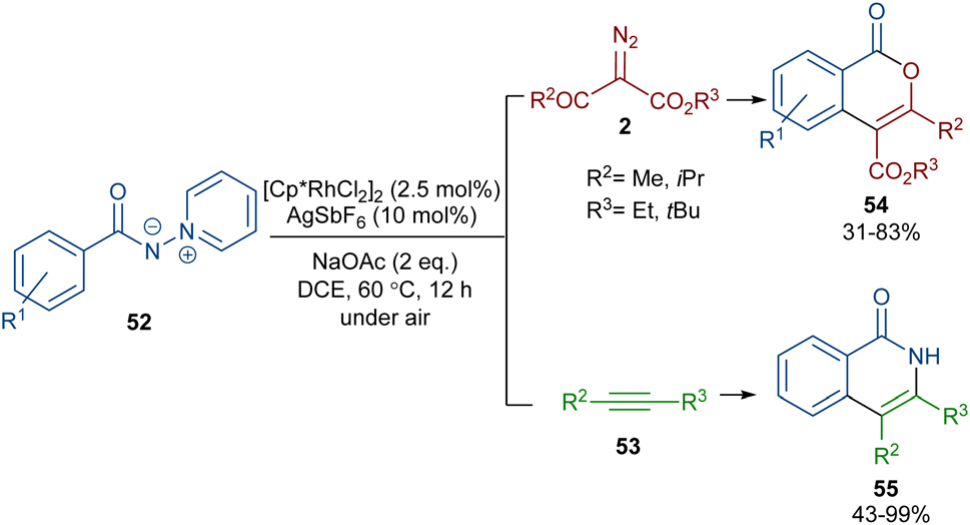
Rh-catalyzed annulation of *N*-iminopyridinium ylides with diazo compounds and alkynes.

**Scheme 28:**
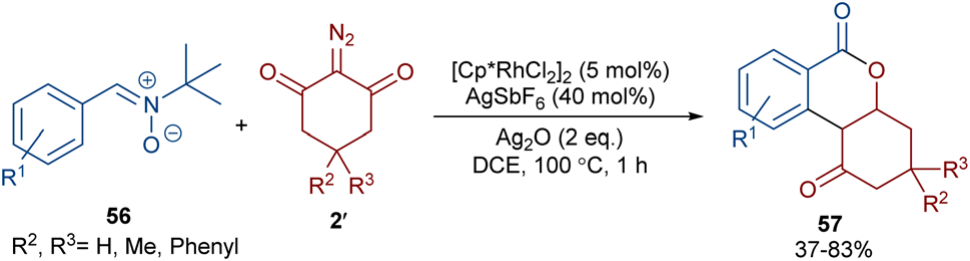
Rh-catalyzed reaction of nitrones and cyclic diazo compounds.

When *N*-iminopyridinium ylides 52 were treated with diazo compounds 2 or alkynes 53 in the presence of a Rh(iii) catalyst, two different products were obtained (Scheme 27).^54^ Isocoumarins 54 were obtained from *N*-iminopyridinium ylides 52 and diazo compounds 2 through Rh-carbene formation, migratory insertion, and lactonization. The *N*-amino pyridine and N_2_ molecules were released in this reaction cycle. The replacement of the alkynes 53 as reactants in the reaction with *N*-iminopyridinium ylides 52 yielded isoquinolones 55. In this reaction, the *N*-iminopyridinium ylide acted as an internal oxidant and directing group. The reaction involved rhodacycle formation, alkyne insertion and proto-demetallation. A similar rhodium catalysis system was utilized for the synthesis of isocoumarins through the reaction of cyclic and acyclic diazo compounds with an enaminone catalyst.^55^ In this work, the enaminone assisted C–H coupling with the diazo compounds. Cyclic diazo compounds 2′ and nitrones 56 could also be used for the synthesis of chromenones (Scheme 28).^56^ A broad range of nitrones bearing electro-donating and halogen groups at the aryl ring were compatible in the reaction with cyclic diazo compounds. Changing the *tert*-butyl to benzyl group attached to the nitrone *N*-atom also yielded the target product in 45% yield. Only nitrones with electron-withdrawing groups (NO_2_ and CO_2_H) and 2-diazo-1,3-indandione were not found to be workable.

**Scheme 29:**
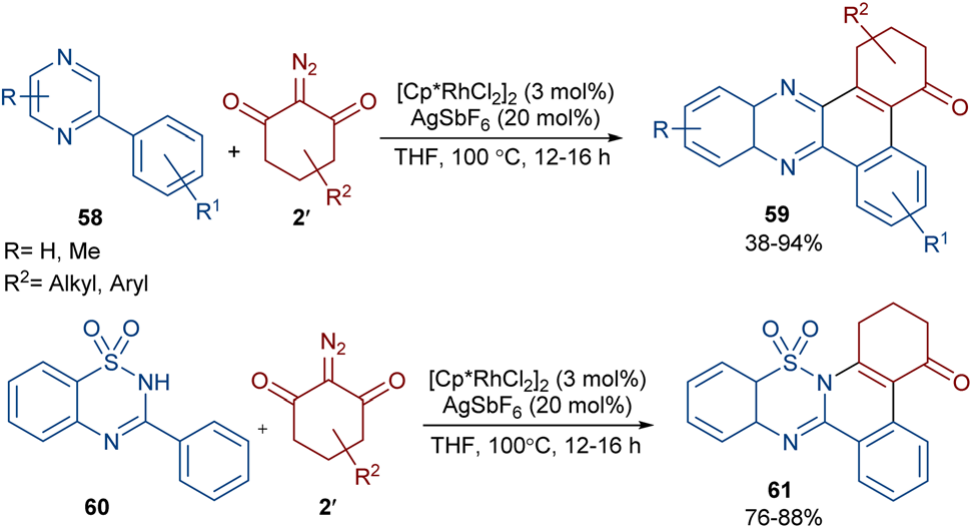
Rh-catalyzed reaction of 2-arylquinoxalines with cyclic 2-diazo-1,3-diketones.

**Scheme 30:**
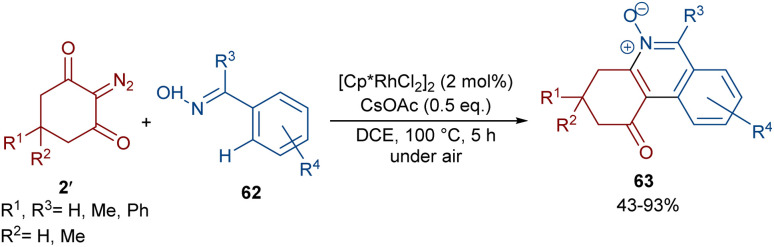
Rh-catalyzed reaction of cyclic 2-diazo-1,3-diones and aryloximes.

Reddy and co-workers reported the annulation of cyclic 2-diazo-1,3-diketones 2′ with 2-arylquinoxalines 58 under rhodium(iii) catalysis (Scheme 29).^57^ A new library of 2,3-dihydrodibenzo[*a*,*c*]phenazin-4(1*H*)-ones 59 and benzo[5,6][1,2,4]thiadiazino[2,3-*f*]phenanthridin-5(6*H*)-one-10,10-dioxides 61 were constructed through nitrogen atom-assisted *ortho* C–H activation of 2-arylquinoxaline with Rh-carbene insertion, migratory insertion of the Rh-carbene into the Rh–C bond, reductive elimination, and dehydration. A similar pathway was postulated in the case of the 3-aryl-2*H*-benzo[*e*][1,2,4]thiadiazine-1,1-dioxide 60 substrates. Additionally, further transformation of the ketone group of the product into the alcohol or oxime moieties was successful. A similar pathway was suggested for the rhodium-catalyzed preparation of fused isoquinoline *N*-oxides 63 from cyclic 2-diazo-1,3-diones 2′ and aryloximes 62 (Scheme 30).^58^ The reaction involved oxime-directed C–H activation in the presence of Rh(iii), carbene insertion, migratory insertion and intramolecular cyclization.

**Scheme 31:**
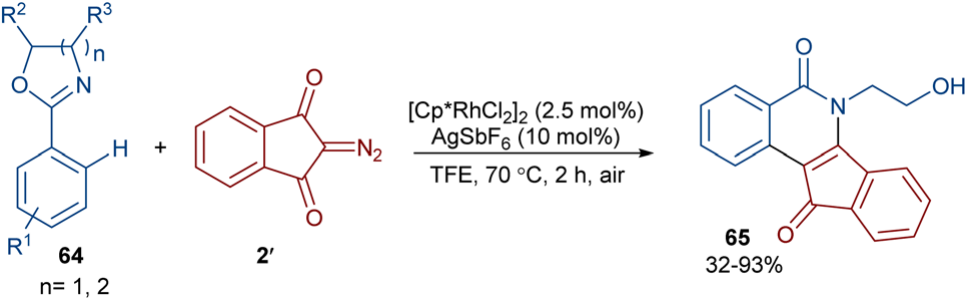
Rh-catalyzed annulation of 2-diazo-1,3-indandiones and 2-phenyloxazolines.

2-Diazo-1,3-indandiones 2′ can efficiently react with 2-phenyloxazolines 64 to construct indenoisoquinolinones 65 in the presence of a rhodium(iii) catalyst (Scheme 31).^59^ This (4 + 2)-annulation reaction proceeded *via* a Rh-carbenoid strategy. Cyclic 1,3-diazo cyclohexanone was also converted to the corresponding product in 72% yield. The gram-scale synthesis of a product (1.29 g, 89%) showed the utility of this method.

**Scheme 32:**
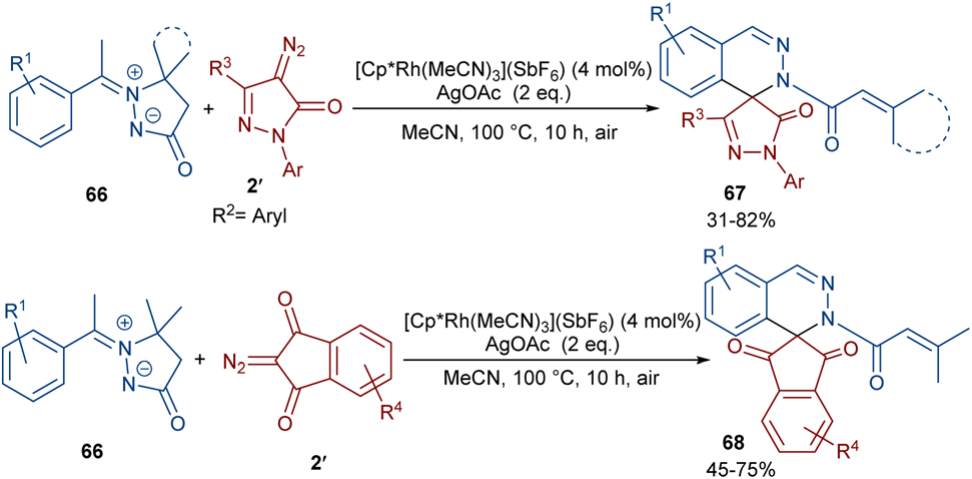
Rh-catalyzed annulation of aryl azomethine with diazo homophthalimides.

**Scheme 33:**
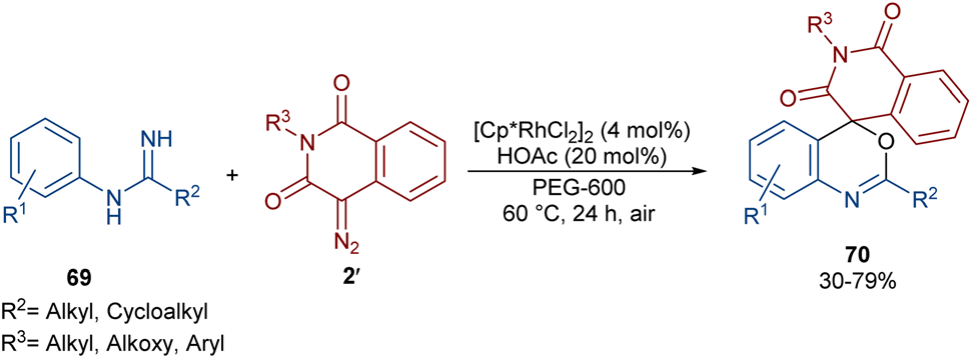
Rh-catalyzed annulation of *N*-aryl amidines with diazo homophthalimides.

**Scheme 34:**
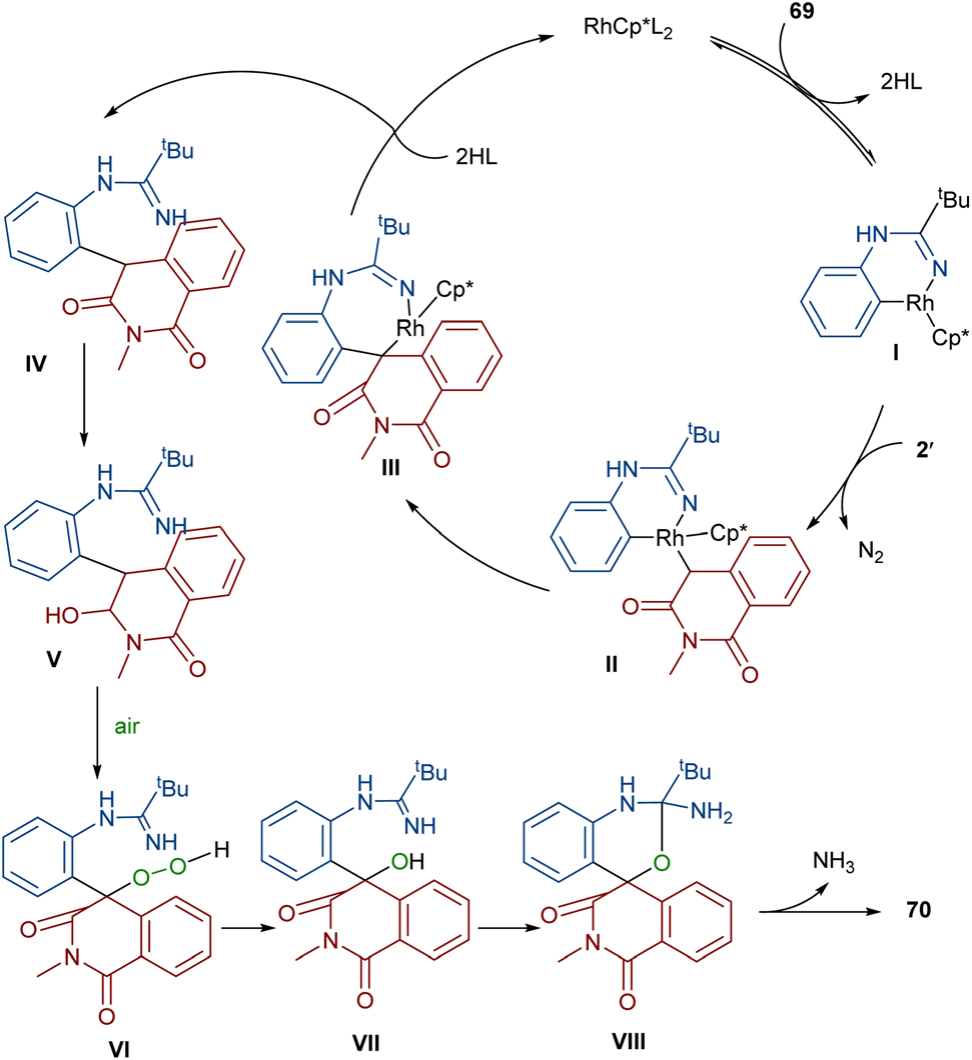
Plausible mechanism for the Rh-catalyzed annulation of *N*-aryl amidines with diazo homophthalimides.

In 2022, Zhang, Fan and co-workers developed the assembly of spirocyclic dihydrophthalazines 67 from aryl azomethine imines 66 with cyclic diazo compounds 2′ (Scheme 32).^60^ The reaction seems to proceed through a Rh-catalyzed azomethine imine-assisted cyclometallation, carbene insertion, isomerization and reductive elimination. Again, this group explained that the synthesis of spiro-isoquinoline from diazo compounds could be achieved through the (4 + 1 + 1)-annulation of *N*-aryl amidines with diazo homophthalimides (Scheme 33).^61^ The reaction features a broad substrate scope, PEG-600 as a sustainable solvent, O_2_ as a green oxidant, and a low reaction temperature. According to the mechanism in Scheme 34, the rhodium-catalyzed amidine-directed *ortho*-C–H bond cleavage of 69 formed the six-membered rhodacycle I, which coordinated with diazo compound 2′ to give Rh-carbene II. The carbene migratory insertion into the Rh–C(sp^2^) bond yielded intermediate III, followed by the protonation to obtain intermediate IV under acidic conditions. Next, the amidine abstracted a benzylic proton from IV to afford enolate V, followed by oxidation and further protonation towards hydroperoxide species VI. In this step, VI reacted with reductant IV to provide alcohol VII, which under an intramolecular nucleophilic attack of the oxygen to the amidine produced intermediate VIII. Finally VIII removed an ammonia molecule to form product 70.

**Scheme 35:**
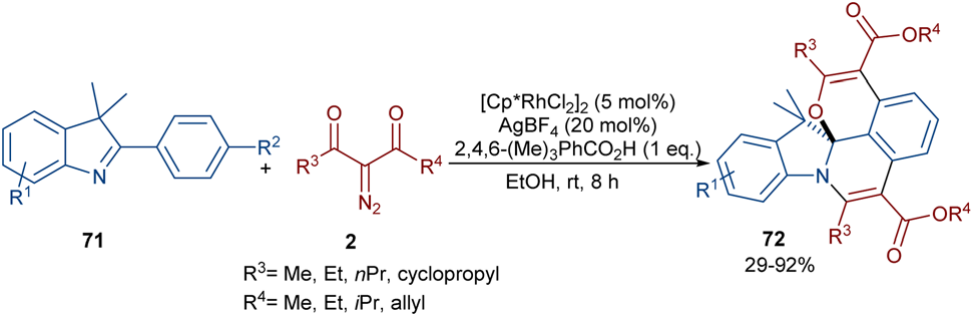
Rh-catalyzed annulation of 2-diazo compounds and 2-phenyl-3*H*-indoles.

**Scheme 36:**
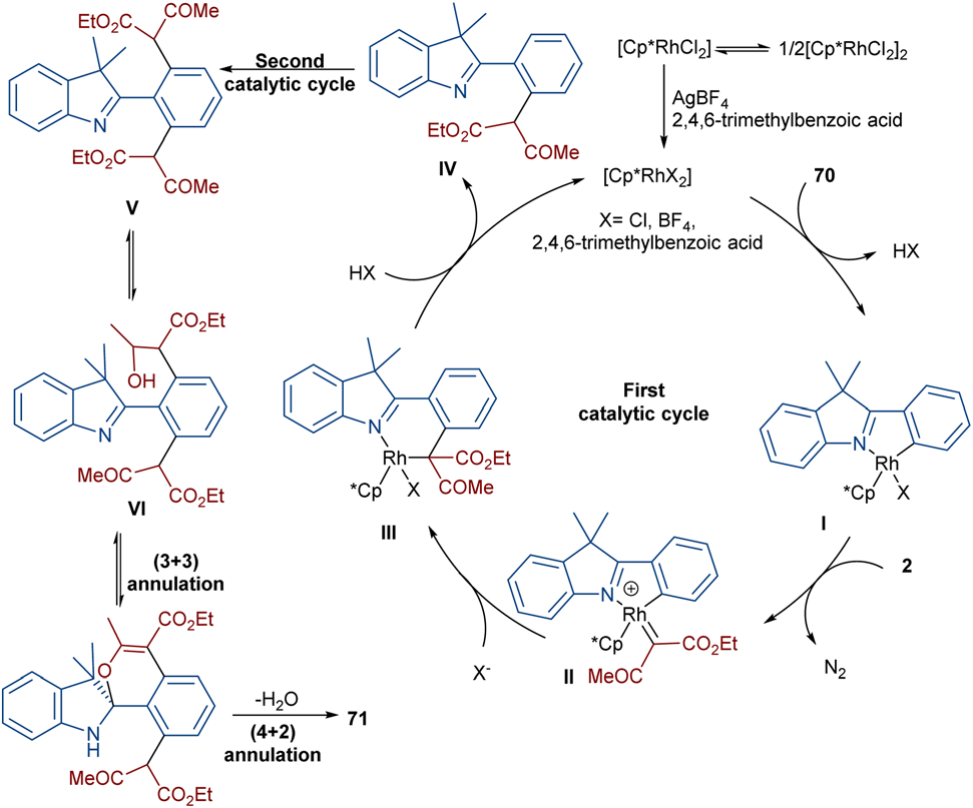
Tentative mechanism for the Rh-catalyzed annulation of 2-diazo compounds and 2-phenyl-3*H*-indoles.

Liu, Zhou and co-workers succeeded in synthesizing a series of highly fused indole heteropolycyclic compounds 71 through an uncommon (3 + 3)- and (4 + 2)-cycloaddition sequence (Scheme 35).^62^ For this purpose, they employed 2-phenyl-3*H*-indoles 70 and diazo compounds 2 as the feedstock in the presence of a rhodium(iii) catalyst. Interestingly, the diazo compound acted as a C3 synthon in the (3 + 3)-cycloaddition and as a C2 synthon in the (4 + 2)-cycloaddition. The authors proposed a tentative mechanism for this transformation involving the initial C(aryl)–H bond activation of 70 Rh(iii) to form the five-membered rhodacycle I, followed by interaction with diazo compound 2 to produce the rhodium carbene II. Subsequent migratory insertion gave the six-membered rhodacycle III, which by further elimination furnished intermediate IV. Sequential C–H activation in IV yielded the disubstituted indole V, which was then converted into enol VI. In this step, the hydroxyl group nucleophilically attacked the imino group to obtain spiro intermediate VII*via* (3 + 3)-cyclization. Ultimately, the NH free indole attacked the carbonyl group to yield product 71 along with H_2_O elimination *via* (4 + 2)-cyclization (Scheme 36).

**Scheme 37:**
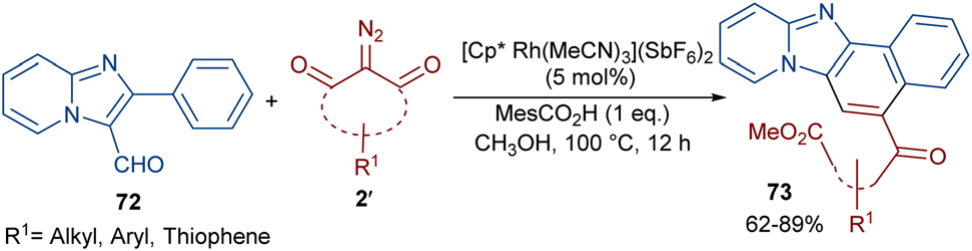
Rh-catalyzed annulation of 2-diazo compounds and 2-arylimidazo[1,2-*a*]pyridines.

**Scheme 38:**
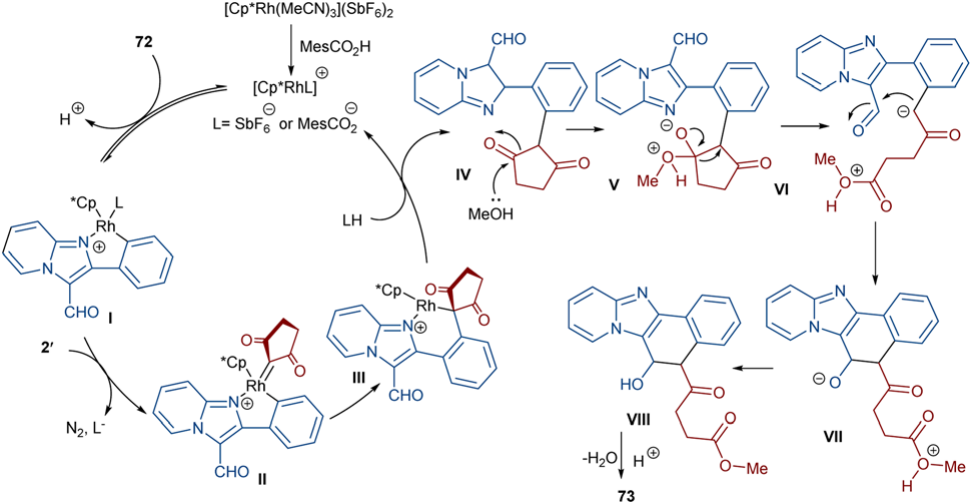
Plausible mechanism for the Rh-catalyzed annulation of 2-diazo compounds and 2-arylimidazo[1,2-*a*]pyridines.

Li *et al.* developed a strategy for the assembly of naphtho[1′,2’:4,5]imidazo[1,2-*a*]pyridines 73 from 2-arylimidazo[1,2-*a*]pyridines 72 and cyclic 2-diazo-1,3-diketones 2′ (Scheme 37).^63^ They proposed a carbene insertion/(5 + 1)-annulation pathway, in which the diazo compounds act as a C1 source. First, an active catalyst [Cp*RhL]^+^ was formed from the ligand exchange between the Rh catalyst and MesCO_2_H. Sequential C–H activation, carbene formation, and migratory insertion resulted in the formation of six-membered rhodacycle III. Then, III underwent protonolysis to give intermediate IV and regenerated [Cp*RhL]^+^. Next, a retro-Claisen reaction occurred with intermediate VI, followed by intramolecular aldol condensation and subsequent dehydrative aromatization under acidic conditions to produce 73 (Scheme 38). The results of the H/D experiments suggested the reversibility of the aryl C(sp^2^)–H activation and the KIE values (*K*_H_/*K*_D_ = 1.2) showed that the C–H activation may not be rate-limiting step.

**Scheme 39:**
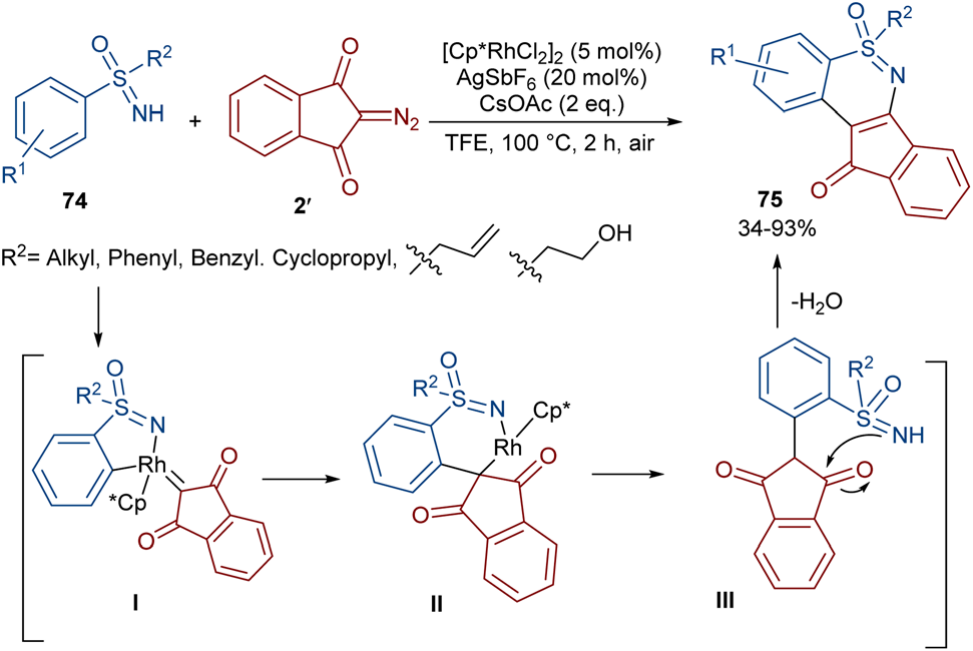
Rh-catalyzed annulation of diazo compounds and sulfoximides.

The formation of *N*,*S*-heterocyclic compounds from diazo indandiones can be achieved under rhodium catalysis (Scheme 39).^64^ Sulfoximide derivatives 74 were utilized as a coupling partner to produce fused tetracyclic indeno-1,2-benzothiazines 75 in up to 93% yield. The reaction involved a five-membered rhodacycle intermediate I, which was added to diazo-indandione 2 to generate the rhodium carbenoid II. Migratory insertion afforded a six membered rhodacycle III, followed by proto-demetallation and dehydration. The gram-scale synthesis of a product (1.35 g, 84%) and the further reduction of the ketone unit to either an alcohol or methylene group demonstrated the utility of this method.

**Scheme 40:**
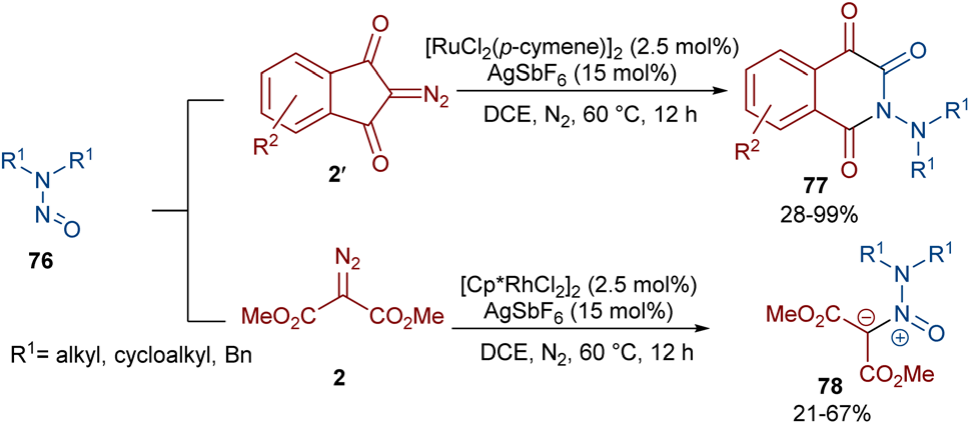
Rh/Ru-catalyzed reaction of cyclic 2-diazo-1,3-diketone, carbodiimide, and 1,2-dihaloethane.

**Scheme 41:**
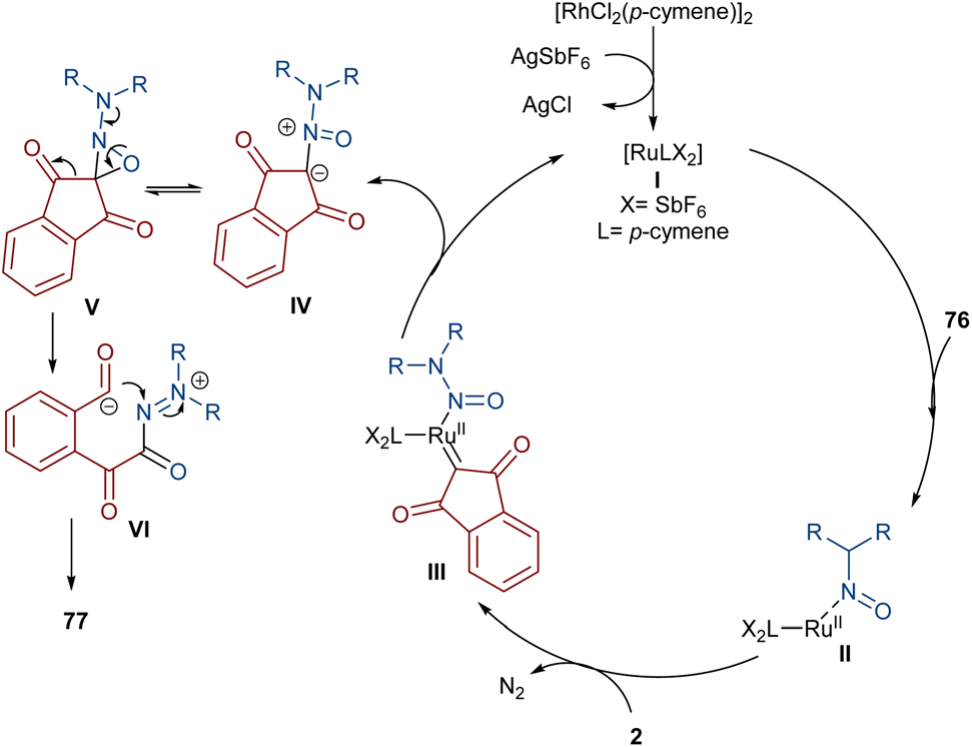
Possible mechanism for the Ru-catalyzed reaction of cyclic 2-diazo-1,3-diketone, carbodiimide, and 1,2-dihaloethane.

Very recently, the Yan research group disclosed two synthetic strategies including diazo compounds (Scheme 40).^65^ They treated diazo compounds 2 and *N*,*N*-dialkylnitrosoamines 76 in the presence of the ruthenium and rhodium catalysts. The method delivered a new library of isoquinoline-1,3,4-trione derivatives 77 using diazoindandiones as a carbene synthon in the presence of ruthenium(ii) complex, while changing the diazo compound and the catalyst into dimethyl diazomalonate and rhodium(iii), respectively, resulted in the formation of nitroso ylide products. The authors proposed a credible mechanism assuming Ru(ii) to be the catalyst. Initially, the active catalyst I was generated from [RuCl_2_(*p*-cymene)]_2_ with AgSbF_6_, and coordinated with *N*,*N*-dialkylnitrosoamine 2 leading to intermediate II, which was then added to diazoindandione 2 to form the ruthenium carbene III with the removal of N_2_. The nitroso ylide IV was obtained from the liberation of the active Ru(ii) catalyst I, followed by conversion to oxaziridine V. Then, the cleavage of the N–O and C–C bonds afforded a stable acylazo cation III, which following an intramolecular cyclization yielded product 77. When dimethyl diazomalonate 78 was used as a substrate the reaction ended during the nitroso ylide VI step. It was found that two esteric groups stabilized this intermediate (Scheme 41).

### 2.3. Synthesis of seven-membered rings

**Scheme 42:**
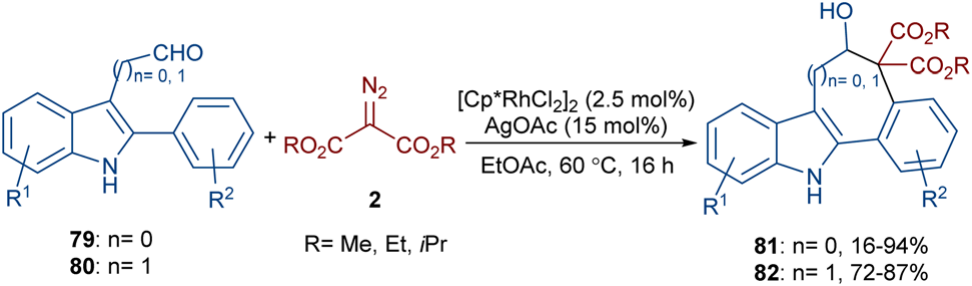
Rh-catalyzed annulation of 3-aldehyde-2-phenyl-1*H*-indoles with diazo compounds.

The annulation of 3-aldehyde-2-phenyl-1*H*-indoles 79 and acetaldehyde-2-phenyl-1*H*-indoles 80 with diazo compounds 2 can lead to 5*H*-benzo[*a*]carbazol-6-ols 81 and benzo[6,7]cyclohepta[1,2-*b*]indol-6-ols 82, respectively (Scheme 42).^66^ In this regard, Hu and co-workers employed a combination of rhodium(iii) and silver(i) salts in the reaction. Although a rational mechanism was not reported by the authors, they proposed a sequence of C–H activation, carbene insertion, and an aldol-type cyclization. A gram-scale experiment, the oxidation of the alcohol to a ketone group and the elimination of ethanol and CO_2_ molecules to give alkenes were also carried out in this work.

**Scheme 43:**
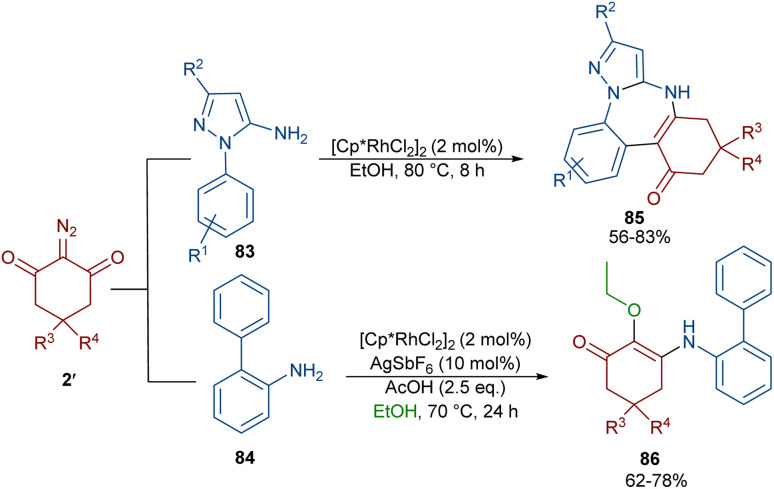
Rh-catalyzed annulation of 1-aryl-1*H*-pyrazol-5-amines with cyclic 2-diazo-1,3-diketones.

Again, Shang and co-workers utilized this Rh complex to assemble benzo[*f*]pyrazolo[1,5-*a*][1,3]diazepines 85 (Scheme 43).^67^ For this purpose, they treated 1-aryl-1*H*-pyrazol-5-amines 83 with cyclic 2-diazo-1,3-diketones 2′ under milder reaction conditions. The reaction proceeded through the Rh-catalyzed pyrazole ring-assisted C–H activation, rhodacycle formation, carbene insertion and intramolecular nucleophilic cyclization. However, the use of [1,1′-biphenyl]-2-amine 84 as a substrate in the reaction with diazo compound 2′ led to the uncyclized product 86. This compound was produced *via* a sequence of 1,1′-insertion, dehydration, and isomerization steps.

### 2.4. Rhodium-catalyzed functionalization reactions of diazo compounds

**Scheme 44:**
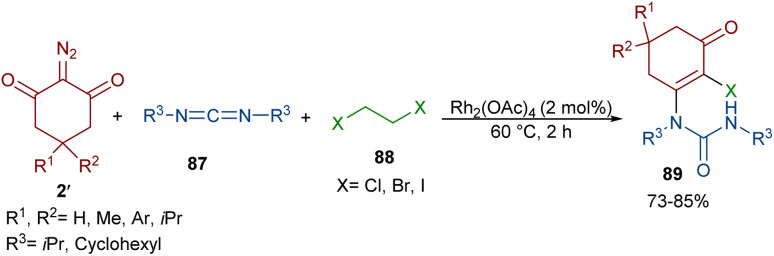
Rh-catalyzed reaction of cyclic 2-diazo-1,3-diketone, carbodiimide, and 1,2-dihaloethane.

**Scheme 45:**
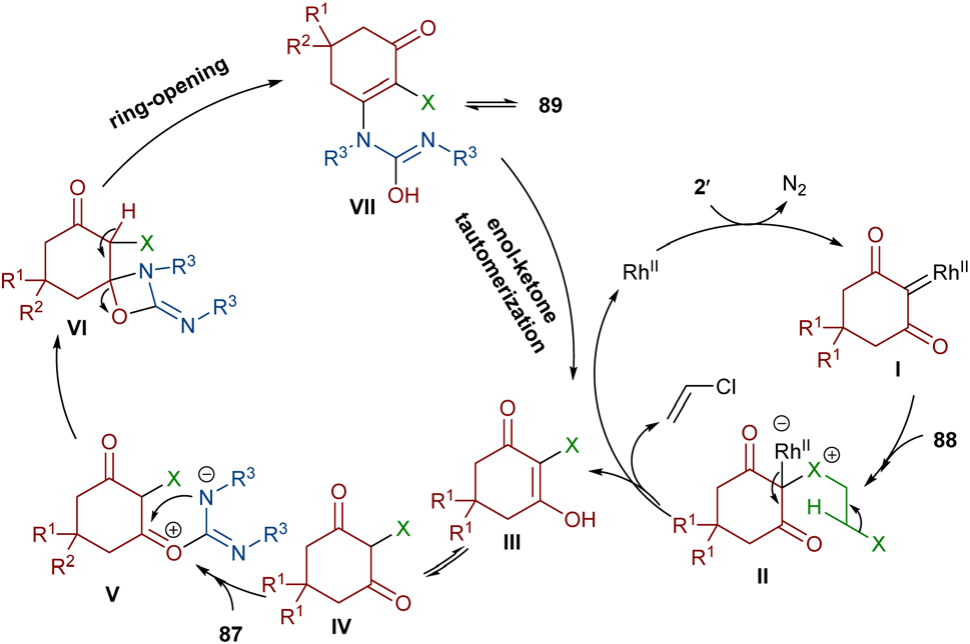
Possible mechanism for the Rh-catalyzed reaction of cyclic 2-diazo-1,3-diketone, carbodiimide, and 1,2-dihaloethane.

An unsymmetrical reaction between cyclic 2-diazo-1,3-diketone, carbodiimide, and 1,2-dihaloethane derivatives was performed in the presence of a rhodium complex (Scheme 44).^68^ In this procedure, urea derivatives were successfully synthesized in good yields. As shown in Scheme 45, the Rh catalyst decomposed diazo compound 2′ into the active Rh-carbene species I, which reacted with 1,2-dihaloethanes 88 to provide halonium ylide II. Through an intramolecular HCl abstraction in II, intermediate III, haloethylene and the Rh(ii) catalyst were formed. Intermediate III can tautomerized with IV. This intermediate was then subjected to a nucleophilic attack at the carbonyl oxygen by the carbon of carbodiimide 87 to provide a carbonyl ylide V. Subsequent intramolecular nucleophilic addition in V gave a four-membered ring intermediate VI, which underwent ring-opening and E2 elimination to give intermediate VII, followed by an enol-ketone tautomerization to access product 89. The utility of the reaction was demonstrated by a gram-scale synthesis of the desired product (1.13 g, 83%).

**Scheme 46:**
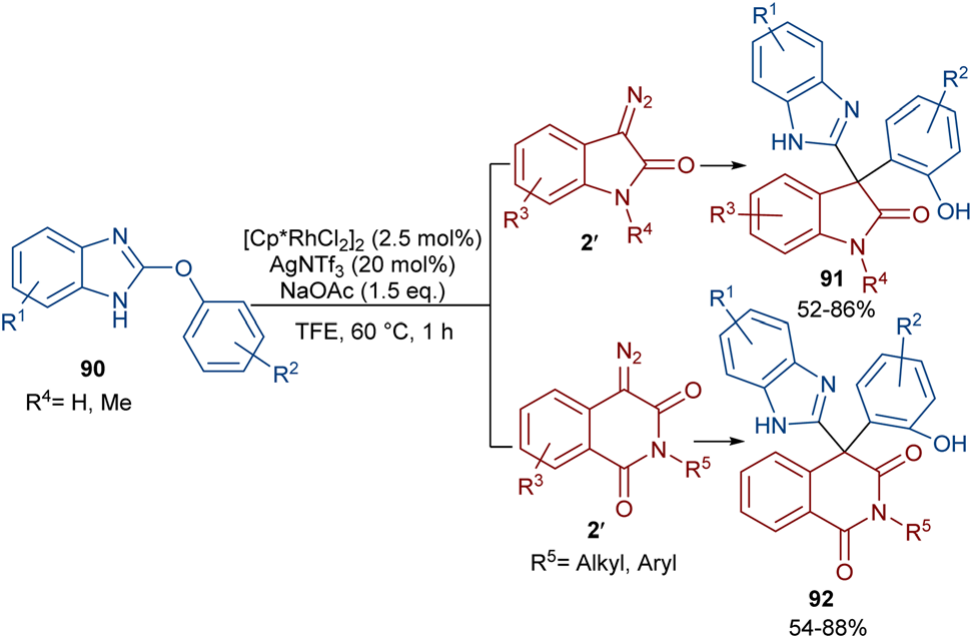
Rh-catalyzed reaction of 2-phenoxy-1*H*-benzo[*d*]imidazole and diazo compounds.

**Scheme 47:**
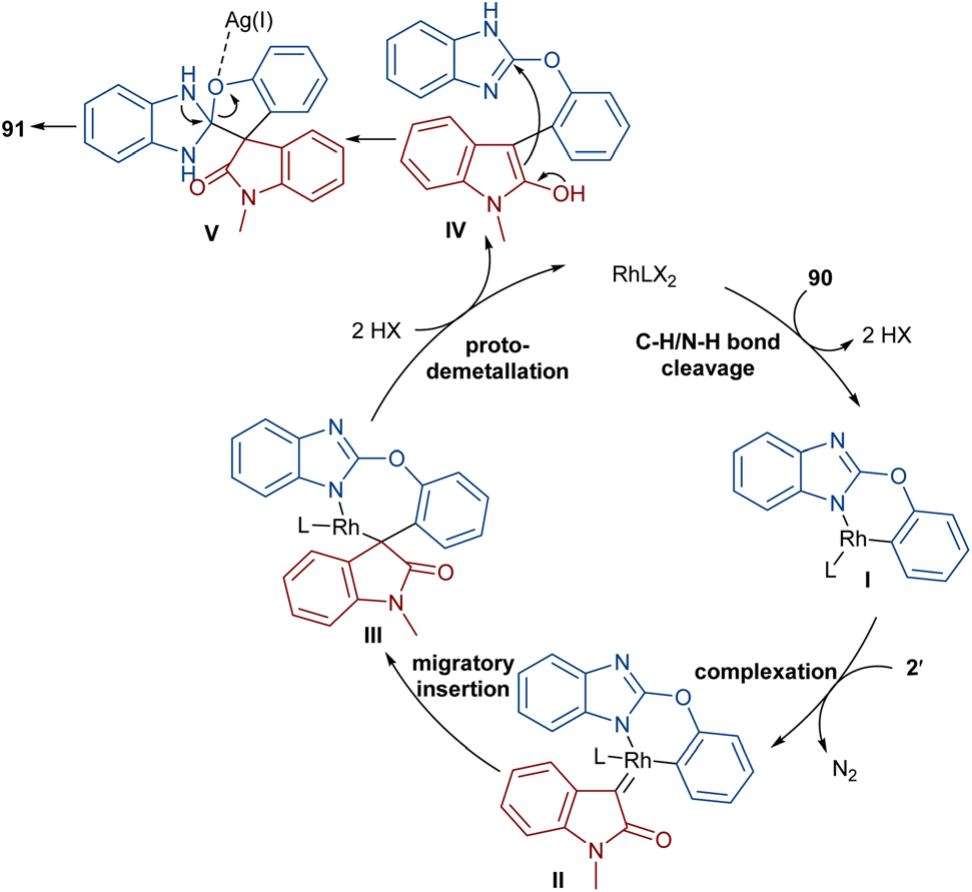
Catalytic cycle for the Rh-catalyzed reaction of 2-phenoxy-1*H*-benzo[*d*]imidazole and diazo compounds.

The reaction of 2-phenoxy-1*H*-benzo[*d*]imidazoles with two different diazo compounds can lead to diaryl oxindole or diaryl isoquinolinedione products (Scheme 46).^69^ In this context, diazo oxindoles or diazo homophthalimides were used as the diazo compounds, respectively. Interestingly, the reaction did not proceed through the expected Rh-catalyzed C–H/N–H bond metallation and formal (5 + 1)-spiroannulation under these conditions, but resulted in the production of the unexpected unsymmetrical 3,3-diaryl oxindole derivatives 91. As shown in the mechanism in Scheme 47, Rh-catalyzed NH-assisted C–H bond activation led to rhodacycle I, which coordinated with 2′ to form Rh-carbene intermediate II. The carbene migratory insertion into the C–Rh bond, and subsequent proto-demetallation produced intermediate IV along with the release of the Rh(iii) catalyst. Then, this cascade process moved through an intramolecular C-nucleophilic addition to form intermediate V, which underwent C–O bond cleavage in the presence of the Lewis acidic Ag(i) to furnish product 91. Moreover, the practicality of this method was shown by the esterification of the alcohol group of the product in 74% and 90% yields and a large-scale synthesis resulting in 1.58 g of product in 89% yield.

**Scheme 48:**
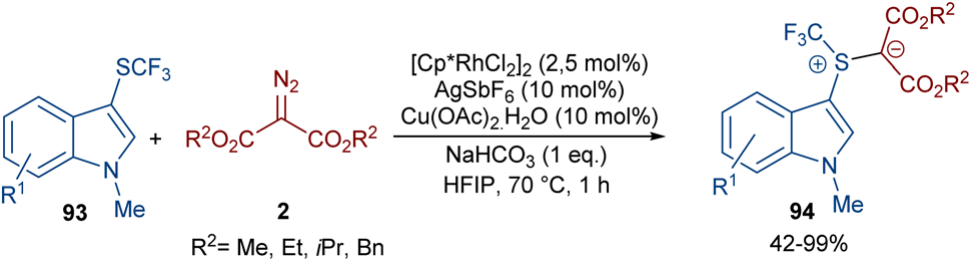
Rh-catalyzed reaction of 1-methyl-3-((trifluoromethyl)thio)-1*H*-indole and diazo compounds.

**Scheme 49:**
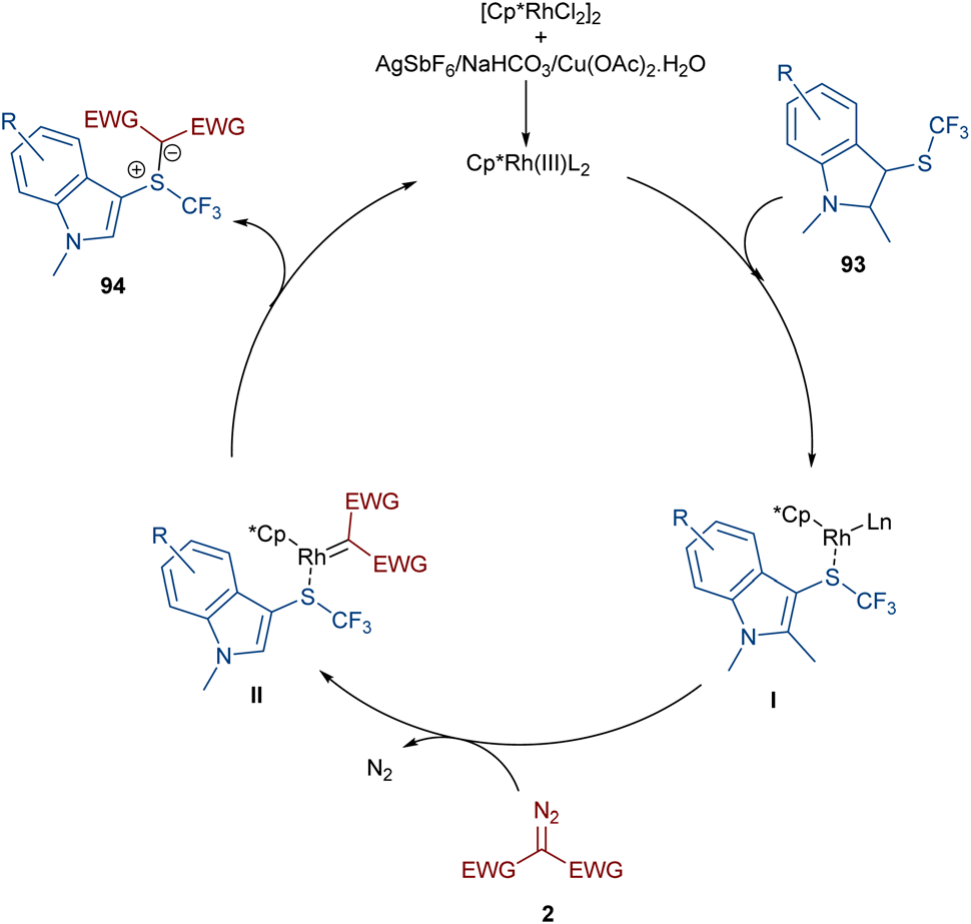
Credible mechanism for the Rh-catalyzed reaction of 1-methyl-3-((trifluoromethyl)thio)-1*H*-indole and diazo compounds.

A rhodium-carbene approach was proposed for the synthesis of a library of indole-substituted trifluoromethyl sulfonium ylides 94 (Scheme 48).^70^ 1-Methyl-3-((trifluoromethyl)thio)-1*H*-indole derivatives 93 reacted with diazo compounds 2 in the presence of a rhodium(iii) pre-catalyst, which was converted to an active Cp*Rh(iii) catalyst I*via* ligand exchange with AgSbF_6_, Cu(OAc)_2_, NaOAc, or NaHCO_3_. The coordination of the sulfur atom of substrate 93 with Rh, and further coordination of diazo compound 2 with the metal center resulted in Cp*Rh(iii)-carbenoid intermediate III together with the expulsion of N_2_. The reductive elimination of III afforded product 94 and regenerated the Cp*Rh(iii) species (Scheme 49). Some substrates, including diazo compounds containing di-*tert*-butyl, and aryl substituents, were not successfully converted, maybe because of steric hindrance with the Rh complex. Also, a trifluoromethyl sulfide group attached the benzyl or phenyl motifs did not work under these conditions.

## 3 Conclusions

As shown in this review, the reactions of diazo compounds using a rhodium catalyst can be carried out *via* a metal-carbene strategy. Both Rh(iii) and Rh(ii) catalysts could efficiently catalyze the coupling reaction/annulations of diazo compounds with other reactants *via* the formation of highly active Rh-carbenoid intermediates. The DGs on the coupling substrates played an important role in selectively directing the C–H insertion by the metal. Although most DGs contained nitrogen groups, the use of substrates with poorer coordinating ability, such as oxygen and sulphur groups, should be further investigated. Despite the remarkable progress in the Rh-carbene strategy, various coupling reactants in the reaction with diazo compounds are still unexplored. Since most synthetic methods are reported under harsh reaction conditions, it would be desirable to develop mild and green conditions using organocatalysts in combination with rhodium. In addition, the use of chiral ligands in combination with rhodium catalysis for the synthesis of enantioenriched bioactive molecules could also be attractive in medicinal chemistry.

## Data availability

All data associated with this manuscript are available within the article.

## Conflicts of interest

There are no conflicts to declare.
